# KDM4B is a coactivator of c-Jun and involved in gastric carcinogenesis

**DOI:** 10.1038/s41419-019-1305-y

**Published:** 2019-01-25

**Authors:** Meng-Chen Wu, Hsin-Hung Cheng, Ta-Sen Yeh, Yi-Chen Li, Tsan-Jan Chen, Wei Yang Sit, Chih-Pin Chuu, Hsing-Jien Kung, Shu Chien, Wen-Ching Wang

**Affiliations:** 1 Institute of Molecular and Cellular Biology and Department of Life Science, National Tsing-Hua University, Hsinchu, 300 Taiwan; 2 Department of Surgery, Chang Gung Memorial Hospital, Taoyuan, 333 Taiwan; 3 Institute of Cellular and System Medicine, National Health Research Institutes, Miaoli, 350 Taiwan; 4 Department of Biochemistry and Molecular Medicine, University of California, Davis, Sacramento, CA 95616 USA; 5 Institute of Molecular and Genomic Medicine, National Health Research Institutes, Miaoli, 350 Taiwan; 6 Institute of Engineering in Medicine, University of California, San Diego, La Jolla, CA 92093 USA

## Abstract

KDM4/JMJD2 Jumonji C-containing histone lysine demethylases (KDM4A–D) constitute an important class of epigenetic modulators in the transcriptional activation of cellular processes and genome stability. Interleukin-8 (IL-8) is overexpressed in gastric cancer, but the mechanisms and particularly the role of the epigenetic regulation of IL-8, are unclear. Here, we report that KDM4B, but not KDM4A/4C, upregulated IL-8 production in the absence or presence of *Helicobacter pylori*. Moreover, KDM4B physically interacts with c-Jun on *IL-8*, *MMP1*, and *ITGAV* promoters via its demethylation activity. The depletion of KDM4B leads to the decreased expression of integrin αV, which is exploited by *H. pylori* carrying the type IV secretion system, reducing IL-8 production and cell migration. Elevated KDM4B expression is significantly associated with the abundance of p-c-Jun in gastric cancer and is linked to a poor clinical outcome. Together, our results suggest that KDM4B is a key regulator of JNK/c-Jun-induced processes and is a valuable therapeutic target.

## Introduction

Histone lysine demethylase 4 (KDM4), which catalyzes the removal of methyl-lysine marks from histone 3, includes four members, KDM4A, KDM4B, KDM4C, and KDM4D^1^. The Jumonji C domain of this family shares a homologous β-jellyroll structure and a conserved active-site region that chelates α-ketoglutarate and Fe(II) for the demethylation of the repressive mark H3K9me3/me2 enrinched in heterochromatic areas^2–7^. Accumulating evidence implicates the overexpressions of KDM4A, KDM4B, and KDM4C in the efficient growth of human malignancies, including breast, colorectal, lung, prostate, and other tumors^1^. Furthermore, KDM4A and KDM4B are often amplified in gastric cancer, neuroblastoma, and ovarian cancer^8–11^. KDM4A regulates chromatin during DNA replication and stem cell genome reprogramming^8,12^. KDM4A can also interact with the co-repressor NCoR to suppress the TRAIL-DR5 pathway^13^ and functions as a key regulator of tumor metabolism via E2F1^14^. KDM4B controls DNA repair and mitochondrial apoptosis, and reprograms the genomes of somatic cells of cloned embryos to control arrest^15–17^. KDM4C regulates pluripotency and embryonic development^18,19^. KDM4A-4C act as coactivators of androgen receptor and estrogen receptor, which are promising epigenetic drug targets^5,20–23^. Although these enzymes share a homologous catalytic JmjC domain, recent evidence suggests non-redundant roles of individual members in regulating distinct transcription programs^24,25^.

Interleukin-8 (IL-8), a chemokine acquired in the tumor microenvironment, recruits suppressive immune cells (myeloid-derived suppressor cells) and may induce epithelial-to-mesenchymal transition (EMT) via autocrine and paracrine mechanisms^26–29^. Notably, an elevated level of IL-8 in gastric cancer is correlated with tumor migration, invasion, and chemosensitivity^30,31^. Substantial increases in IL-8 can be triggered by LPS, cytokines, hypoxia, pathogens, and other environmental stresses, and these increases are mediated by transcription factors, including NF-κB and activator protein 1 (AP-1)^29,32^. In the presence of the prominent stomach pathogen *Helicobacter pylori*, NF-κB and AP-1 are translocated into the nucleus, followed by the recruitment of CREB-binding protein/P300 for the synergistic transactivation of IL-8^32,33^. Notably, *H. pylori* strains that carry the *cagA* pathogenicity island encoding the type IV secretion system and an oncoprotein (CagA) are associated with more severe clinical sequelae^34,35^. Translocated CagA perturbs host signaling pathways, leading to inflammation, altered physiology, and genetic/epigenetic changes, and prompting the neoplastic transformation of gastric epithelial cells^36,37^. Infection with CagA-positive *H. pylori* is associated with the highly upregulated expression of IL-8 in a cholesterol-dependent manner^38–40^. However, little is known about the mechanism of initial removal of the repressive histone mark by epigenetic modifiers.

In this study, we examined whether IL-8 production is controlled by a KDM4 member. We showed that KDM4B, rather than KDM4A/KDM4C, significantly activated the production of IL-8 at the transcriptional level in the absence or presence of *H. pylori* challenge. We demonstrate that KDM4B is a coactivator of c-Jun to regulate the expressions of IL-8, MMP1, and integrin αV. The silencing of KDM4B or pharmacological inhibition of c-Jun strongly inhibits the production of IL-8. Thus, our results reveal a novel function of KDM4B in controlling the JNK/c-Jun-induced IL-8-IL-8R axis in gastric cancer, and offer a new strategy in cancer therapy.

## Materials and methods

### Bacteria and cell culture

*H. pylori* 26695 (ATCC 700392) was used as the reference strain in this study. *H. pylori* was routinely cultured on brucella agar plates contained with 2.8% Brucella powder (Becton Dickinson, Franklin Lakes, NJ, USA), 0.2% β-cyclodextrin (Sigma-Aldrich, St. Louis, Missouri, USA), 0.1% yeast extract, 1.5% agar (Cleveland, OH, USA), 1% isovitalex (Becton Dickinson, Franklin Lakes, NJ, USA), and 10% sheep blood in a microaerophilic atmosphere (5% O_2_, 10% CO_2_, and 85% N_2_) at 37 °C for 2 days. The isogenic *H. pylori* mutant *cagA* knockout strain (∆CagA) was constructed as described^41^. AGS cells (ATCC number: CRL-1739), the human gastric adenocarcinoma cell line, were cultured in Ham’s F-12K medium (Thermo, Waltham, MA, USA) contained with 10% fetal bovine serum (Hyclone, Logan, UT, USA) at 37 °C in 5% CO_2_. MKN45 cells (JCRB number: JCRB0254), the human gastric adenocarcinoma cell line, were cultured in RPMI 1640 medium (Thermo, Waltham, MA, USA) contained with 10% fetal bovine serum. 293T cells (ATCC number: CRL-3216), the human embryonic kidney cell line, were cultured in DMEM medium (Thermo, Waltham, MA, USA) contained with 10% fetal bovine serum.

### Antibodies and reagents

Rabbit anti-KDM4A, anti-ERK1/2, anti-p-ERK1/2 (T202/Y204), anti-c-Jun, and anti-p-c-Jun (S63) were purchased from Cell Signaling Technology (Danvers, MA, USA). Rabbit anti-KDM4B was purchased from Bethyl Laboratories (Montgomery, TX, USA). Rabbit anti-KDM4C was purchased from Novus Biological (Littleton, CO, USA). Rabbit anti-H3K9me3 was purchased from Active Motif (Carlsbad, CA, USA). Rabbit anti-IgG, and anti-p-Tyrosine were purchased from Millipore (Billerica, MA, USA). Mouse anti-CagA, and anti-IgG were purchased from Santa Cruz Biotechnology (Dallas, TX, USA). Mouse anti-β-Actin was purchased from Sigma. Lentiviral vector pLKO-control (pLKO), pLKO-shKDM4A (sh4A#1, TRCN0000234912; sh4A#2, TRCN0000234914), -shKDM4B (sh4B#1, TRCN0000018016; sh4B#2, TRCN0000379460), -shKDM4C (sh4C#1, TRCN0000235047; sh4C#2, TRCN0000235048) plasmids were purchased from The RNAi Consortium (TRC).

### Establishment of KDM4B-knockdown cells

Lentivirus particles were produced in 293T cells using pLP1, pLP2, and pLP/VSVG packaging system (Thermo, Waltham, MA, USA) according to the user’s manual. KDM4-knockdown cell lines were established in AGS and MKN45 cells using pLKO, sh4A, sh4B, and sh4C lentivirus infection, and cells were then selected with puromycin (2 μg/ml for AGS and 5 μg/ml for MKN45) for 1 week. Knockdown efficiency was confirmed using qRT-PCR and/or immunoblotting analysis.

### Human cytokine array

The supernatants of pLKO and sh4B#1 AGS cells infected with *H. pylori* at an moi of 50 for 6 h were collected. Human cytokine array Panel A (R&D Systems, Minneapolis, MN, USA) was used to detect cytokines in the supernatants according to the manufacturer’s instructions.

### Quantitative real-time PCR (qRT-PCR)

Total RNAs were extracted with TRIzol reagent (Thermo) and the cDNAs were prepared using the SuperScript III Reverse Transcriptase (Thermo), dNTP (Genedirex, Las Vegas City, NV, USA), and random primers (Thermo). The amount of cDNA was detected with SensiMix™ SYBR® Hi-ROX Kit (Bioline, Taunton, MA, USA) and primers (listed in Table S3) with ABI StepOnePlus Real-Time PCR System (Thermo). All data were triplicated, and normalized to GAPDH.

### Human IL-8 ELISA

AGS cells were infected with *H. pylori* at an moi of 50 for 6 h, and MKN45 cells were infected at an moi of 50 for 3 h. Supernatants were collected to detect IL-8 production by using Human IL-8 Cytoset Kit (Thermo).

### Cell elongated hummingbird phenotype

AGS cells (2×10^5^) were cultured in 6-well plates and then infected with wild-type *H. pylori* at an moi of 50 for 6 h as previously described^39^. Cells were examined with a Zeiss Axio Observer inverted microscope (Carl Zeiss, Jena, Germany) and images were captured and processed by the AxioVision software. The elongated phenotype was defined as cells that had thin needle-like protrusions that were > 20 μm long and had needle-like elongated cells^39^. Over 300 cells were calculated in triplicate experiments.

### Immunoblotting assay

Cells were infected with *H. pylori* at an moi of 50. Cells were washed three times with PBS and cell pellets were collected by a scraper and lysed in RIPA buffer supplemented protease inhibitor (Santa Cruz) and PhosSTOP (Sigma). Appropriate amounts of lysates were fractionated by SDS-PAGE and then transferred onto PVDF membrane (PALL, Port Washington, NY, USA). Membrane was incubated with appropriately diluted primary antibody at 4 °C overnight and then probed with secondary antibodies conjugated with fluorescence. Odyssey Infrared Imaging System (LI-COR Biosciences, Lincoln, NE, USA) was used to detect the level of fluorescence.

### Immunoprecipitation (IP) assay

Cell pellets were collected from 80% confluent wild-type AGS cells and lysed in IP lysis buffer (50 mM Tris-HCl (pH 7.4), 150 mM NaCl, 0.5% NP40) supplemented with protease inhibitor (Santa Cruz). Appropriate amounts of protein samples were incubated with the corresponding antibodies (1 μg) as indicated and 10 μl of PureProteome protein A/G magnetic beads (Millipore) at 4 °C overnight. The beads were washed three times with IP-wash buffer (137 mM NaCl, 2.7 mM KCl, 10 mM Na_2_HPO_4_, 1.8 mM KH_2_PO_4_, 0.1% Tween 20, pH 7.4) and eluted by the IP-lysis buffer, followed by immunoblotting assay with corresponding antibodes as indicated.

### Luciferase activity assay

AGS cells were seeded onto 12-well plates overnight, and then transfected with IL-8, ∆AP-1, or ∆NF-κB luciferase reporter constructs and internal control reporter β-galactosidase using Lipofectamine 2000 (Thermo). After 16-h incubation, cells were infected with *H. pylori* at an moi of 50 for 6 h. The luciferase activities were determined using the Beta-Glo/One-Glo Assay System (Promega Corporation, Madison, WI, USA).

### ChIP assay

AGS cells were infected with *H. pylori* at an moi of 50 for 3 h, and were crosslinked with 1% formaldehyde for 10 min and then quenched in 0.125 M glycine. The crosslinked chromatin was fragmented by sonication. The lysate were subjected to IP using corresponding antibodies as indicated (anti-KDM4B, anti-c-Jun, anti-H3K9me3, and rabbit IgG) and Magna ChIP Protein G Magnetic beads (Millipore) at 4 °C overnight. The ChIP complexes were eluted by elution buffer [50 mM Tris-HCl (pH 8.0), 10 mM EDTA, 1% SDS] and quantified by qRT-PCR (primer sequences listed in Table S3). Fold enrichment was calculated by the ∆∆Ct method.

### Small interfering RNA (siRNA) transfection

To downregulate the expression of *ITGAV* and *ITGB1*, transfection of specific si-RNA (Dharmacon, Lafayette, CO, USA) was performed by using Lipofectamine 2000 according to the manufacturer’s protocol. Expression of *ITGAV* and *ITGB1* was evaluated using qRT-PCR. si-RNA sequences listed in Table S4.

### Microarray

Global expression analysis was performed in AGS cells (pLKO vs. sh4B#1) non-infected or infected by *H. pylori* at an moi of 50 for 6 h (GEO: GSE107703). Microarray analysis was conducted by the National Health Research Institutes, using Affymetrix GeneChip Human Gene 2.0 ST Array (Affymetrix, Santa Clara, CA, USA). Subsequent data analysis was done by DAVID Bioinformatics database. AP-1 and NF-κB binding genes were analyzed by UCSC genome browser tool (http://www.genome.ucsc.edu/).

### Micro-western array (MWA)

Cytokine production analysis in non-infected and *H. pylori*-infected AGS cells (pLKO vs. sh4B#1) was performed by the MWA Core facility of the National Health Research Institutes, using appropriate antibodies as indicated and followed the MWA protocol previously described^42^.

### Wound healing assay

AGS cells were seeded onto 6-well plates overnight. A linear wound was generated by scratching a 10-μl pipette tip onto cells, followed by three washes of PBS to remove the detached cells. Cells were then non-infected or infected with *H. pylori* at an moi of 50. The wound closure was monitored with a Leica microscopy (Leica, Wetzlar, Germany) at indicated time points. The percentage of wound closure was quantified by ImageJ.

### Immunohistochemistry (IHC)

Two consecutive paraffin-embedded human gastric cancer biopsies (*n* = 87) containing paired tumor and adjacent normal tissue were obtained from Chang Gung Memorial Hospital, Taoyuan, Taiwan. IHC staining was performed using anti-KDM4B and anti-p-c-Jun. IHC data for KDM4B and p-c-Jun staining was graded into positive and negative categories based on the presence of strong nuclear staining or not. This study is approved by Institutional Review Board of Chang Gung Memorial Hospital.

### Kaplan–Meier plotter survival analysis

Overall survival and first progression were analyzed using KM Plotter online tool (http://kmplot.com/analysis/) in which the database consists of 1065 gastric cancer patients^43^. Datasets used to examine the relationship with *KDM4B* expression include 212492_s_at, 215616_s_at, 216023_at, and 212495_at and those with *IL-8* expression are 211506_s_at and 202859_x_at.

### Statistical analysis

The Student’s *t*-test was used to calculate the statistical significance of the experimental results between two groups (significance at *p* < 0.05). All values reflect mean ± SD of data obtained from at least three independent experiments. Chi-square test was performed to compare groups with categorical variables in IHC analysis.

## Results

### KDM4B, but not KDM4A/KDM4C, upregulated IL-8 production, particularly upon *H. pylori* infection

**Fig. 1:**
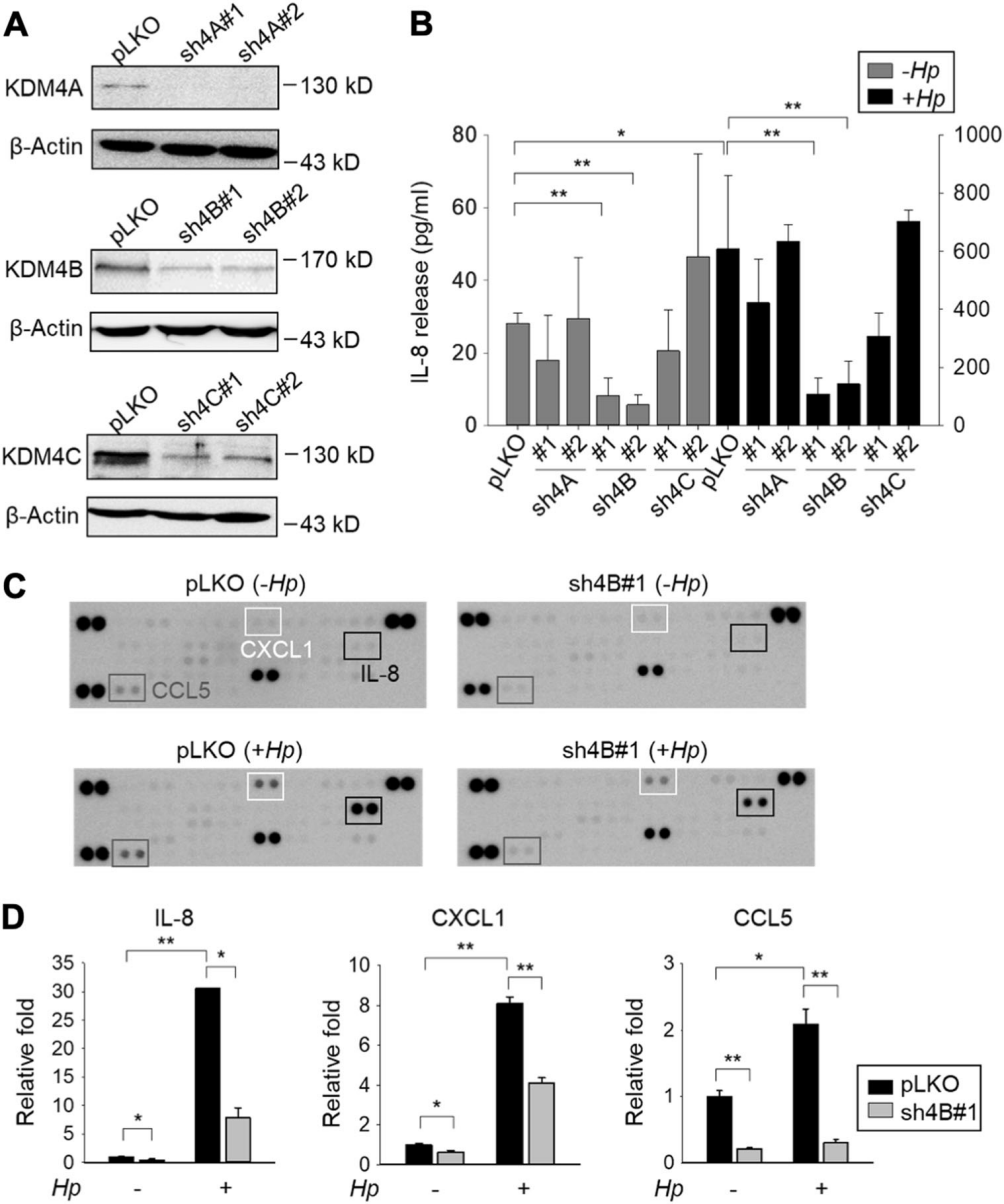
Silencing of KDM4B but not KDM4A/KDM4C inhibits the production of IL-8. **a** Generation of KDM4A-, KDM4B-, and KDM4C-knockdown AGS cells. Cells were infected with lentivirus using a control shRNA (pLKO) and two independent short-hairpin constructs to generate shKDM4A (sh4A#1 and sh4A#2), shKDM4B (sh4B#1 and sh4B#2), and shKDM4C (sh4C#1 and sh4C#2), respectively. Depletion of KDM4A, KDM4B, or KDM4C was confirmed by western blotting analysis. β-Actin was the internal control. **b** IL-8 release was measured from the supernatants of non-infected or *H. pylori*-infected cells by ELISA. Data represent the mean ± standard deviation (SD) from three independent experiments. **c** Images of cytokine arrays for the detection of the relative levels of cytokines from supernatants of pLKO and sh4B#1 cells, respectively. Cells were non-infected (−*Hp*) or infected with *H. pylori* (+ *Hp*) at an moi of 50 for 6 h. **d** The relative fold of IL-8, CXCL1, and CCL5 was measured and shown as a ratio of pLKO (−*Hp*) levels from (**c**). *p < 0.05; **p < 0.01

It is now well recognized that histone modifications are crucial platforms in modulating chromatin conformation and gene transcription. Among the histone demethylases, KDM4 family plays a major role in transcriptional activation^1,44^. We hypothesized that a KDM4 family member regulates the production of IL-8. To test this notion, AGS cells, a gastric cancer cell line, were transduced with control (pLKO) or two independent knockdown constructs (sh4A#1, sh4A#2, sh4B#1, sh4B#2, sh4C#1, and sh4C#2) based on the lentivirus approach to deplete endogenous KDM4A, KDM4B, and KDM4C, respectively (Fig. 1a). Upon *H. pylori* (strain 26695) infection, pLKO cells displayed significantly higher production of IL-8 than did non-infected cells (28.2-fold, *p* < 0.05), consistent with previous results indicating that *H. pylori* stimulates IL-8 production in AGS cells^39^. Of KDM4-knockdown lines, a significantly reduced signal of IL-8 was detected for two KDM4B-knockdown lines, but not for KDM4A- or KDM4C-knockdown lines without *H. pylori* infection (Fig. 1b, *p* < 0.01). With *H. pylori* infection, KDM4B-knockdown, but not KDM4A-/KDM4C-knockdown lines, again showed significantly lower IL-8 production than did the infected pLKO control cells (*p* < 0.01). To test whether KDM4B serves as a general regulator of IL-8 production for gastric cancer cells infected with *H. pylori*, we compared the production of IL-8 in various gastric cancer lines including AGS, MKN28, MKN45, and SNU601. Figure S1A shows that all cell lines had an increased level of IL-8 production upon *H. pylori* infection. Analysis of KDM4B expression revealed that MKN45 had a comparable level with that of AGS (Figure S1B). We thus further evaluated whether the depletion of KDM4B in MKN45 affected the production of IL-8. Upon *H*. *pylori* infection, KDM4B-knockdown MKN45 cells indeed led to a significantly reduced level of IL-8 production, suggesting that KDM4B as an important regulator of IL-8 expression in gastric cancer cells (Figure S1C‒1D).

We next examined the expression levels of 37 cytokines in cell culture supernatants of KDM4B-knockdown and pLKO cells uninfected or infected with *H. pylori*. The levels of IL-8, CCL5, and CXCL1 were significantly downregulated in uninfected KDM4B-knockdown cells. Infection with *H. pylori* further stimulated the production of these cytokines. Notably, IL-8 was the most highly upregulated cytokine (30.6-fold) (Fig. 1c, d). These results suggest that KDM4B is a key epigenetic regulator of IL-8 production, and its effects are even more pronounced upon *H. pylori* infection.

### KDM4B regulates the expression of NF-κB and AP-1 target genes

To characterize the molecular mechanisms by which IL-8 is upregulated in response to KDM4B, differential gene expression between pLKO and KDM4B-knockdown AGS cells was examined by microarray analysis (≥ 2-fold alterations) (GEO: GSE107703). Functional annotations indicated that a number of downregulated genes in uninfected cells were involved in the following functions: response to stimulus, virus, wounding, chemical stimulus, and multi-organism process (Figure S2A). Infection with *H. pylori* revealed the following categories of differentially expressed genes: response to virus, multi-organism process, and response to stimulus. Interestingly, a substantial proportion of downregulated genes (144/300 = 48%) in the non-infected set were also observed in the infected set (Figure S2B). We surveyed whether these genes share the NF-κB and AP-1 binding motifs using the tool provided by the UCSC Genome Browser. Of these genes (*n* = 144), a high fraction (38.2%) showed AP-1 or NF-κB-binding motifs, i.e., 31 had an AP-1-binding site, 18 had an NF-κB-binding element, and 6 (including *IL-8* and *MMP1*) carried both motifs (Figure S2C).

### JNK/c-Jun signaling is crucial for KDM4B-mediated IL-8 production

**Fig. 2:**
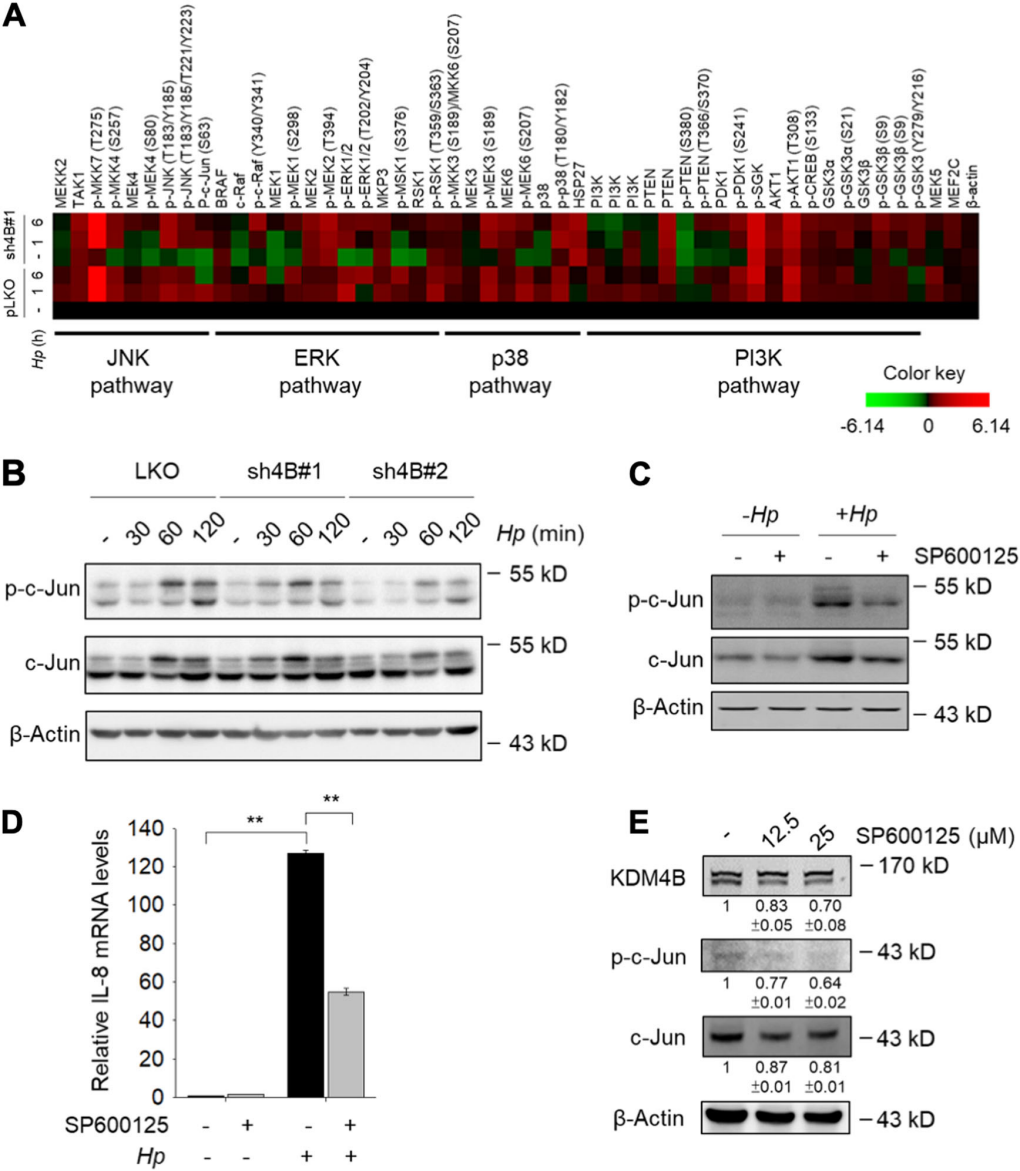
Silencing of KDM4B reduced the JNK/c-Jun and ERK signaling. **a** Micro-Western Array heat-map chart shows the abundance and phosphorylation signal protein fold change of non-infected or *H. pylori*-infected sh4B#1 cells as compared to pLKO control cells. β-Actin was the internal control. **b** The expression of c-Jun and phosphorylation of c-Jun in non-infected or *H. pylori*-infected pLKO, sh4B#1, and sh4B#2 cells were detected by immunoblotting. **c** AGS cells were untreated or treated with SP600125 (25 μM) in the absence (−*Hp*) or presence of *H. pylori* (+ *Hp*) at an moi of 50 for 6 h. c-Jun, p-c-Jun, and β-actin were detected by immunoblotting. **d** IL-8 mRNA levels were measured from AGS cells untreated or treated with SP600125 according to (**c**). **e** AGS cells were treated with indicated concentrations of SP600125, followed by immunoblotting analysis with anti-KDM4B, anti-p-c-Jun, anti-c-Jun, and β-actin antibodies, respectively. *p < 0.05; **p < 0.01

To characterize the signaling pathways involved in KDM4B-mediated IL-8 production, we compared protein abundance and modifications using a western microarray with 59 antibodies (phosphosites) against proteins in four signaling pathways (JNK, ERK, p38, and PI3K). Infected pLKO cells showed stimulated signaling in the four pathways as compared with the levels in non-infected pLKO cells^38,45–47^. For KDM4B-knockdown cells, interestingly, non-infected cells had greatly decreased signals for the JNK (p-JNK and p-c-Jun) and ERK (p-MEK1 and p-ERK1/2) pathways as compared with those of control cells particularly the JNK pathway. The p38 and PI3K pathways exhibited inconsistent and relatively little differences between KDM4B-knockdown and control cells without *H. pylori* infection. Upon *H. pylori* infection, ERK and JNK signaling were only slightly induced in KDM4B-knockdown cells, whereas the p38 and PI3K pathways were essentially unchanged in comparison with the levels (Fig. 2a). The increased level of p-c-Jun was confirmed in infected pLKO cells, but much less in KDM4B-knockdown cells at 60 min and 120 min post-infection by western blotting (Fig. 2b). Treatment with a specific JNK inhibitor SP600125 that blocks the phosphorylation of c-Jun^48^ significantly reduced the expression of *IL-8* in response to *H. pylori* stimulation in AGS cells (Fig. 2c, d) and in MKN45 cells (Figure S3A). Fig. 2e and Figure S3B show that SP600125-treated cells (0–25 μM) exhibited a reduced signal of p-c-Jun and a slightly reduced expression of KDM4B in a dose-dependent manner. These results together suggest that the JNK pathway contributes to KDM4B-mediated IL-8 production.

### KDM4B interacts with c-Jun and regulates *IL-8* promoter activity

**Fig. 3:**
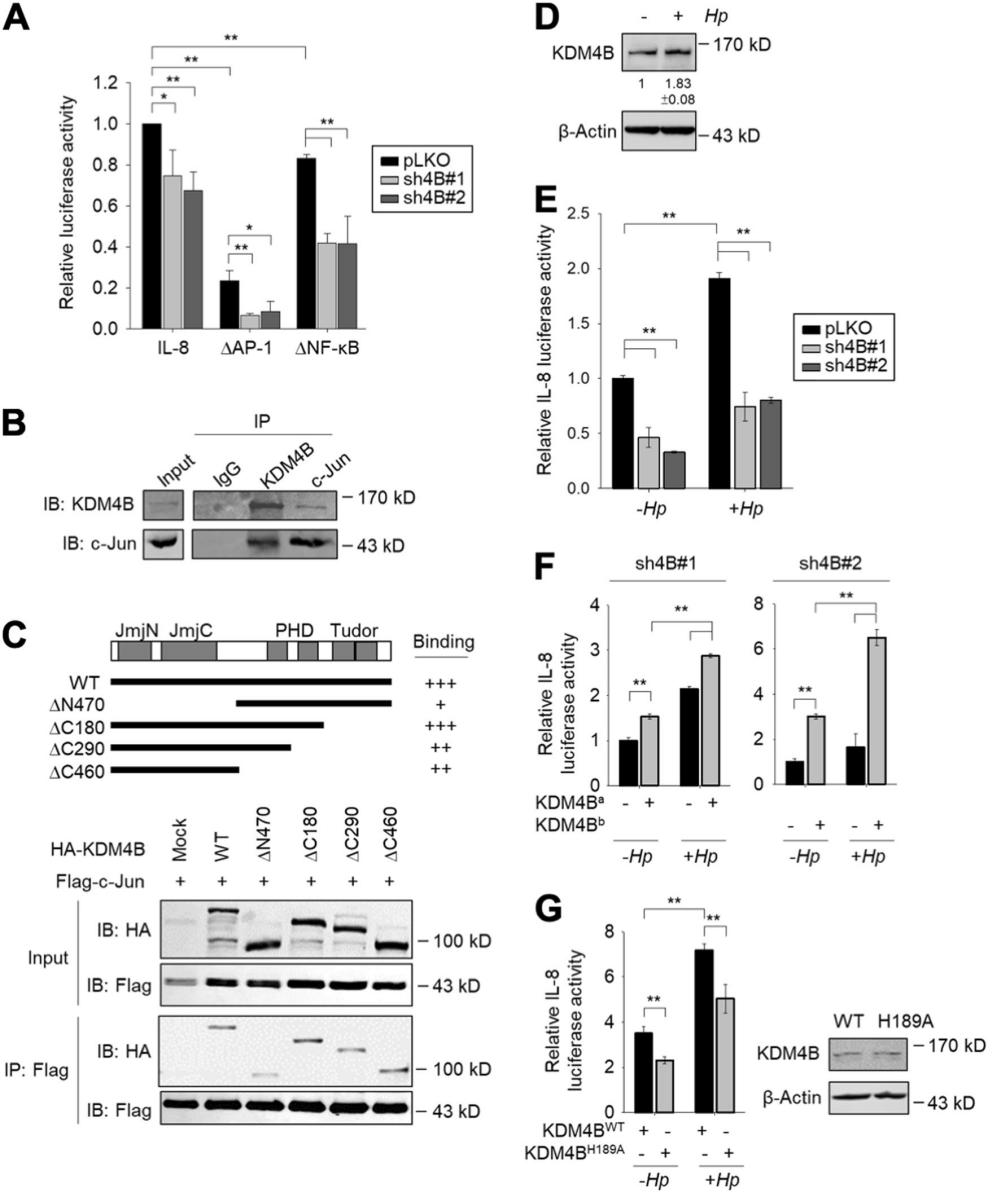
KDM4B interacts with c-Jun and regulates *IL-8* promoter activity. **a** The *IL-8* promoter activity of cells (pLKO, sh4B#1, or sh4B#2) cotransfected with *IL-8*-luc, ∆AP-1, or ∆NF-κB plus β-galactosidase (an internal control). **b** Endogenous association of KDM4B and c-Jun. AGS cell lysates were used for KDM4B and c-Jun IP, respectively. The input and IP were detected by anti-KDM4B or anti-c-Jun antisera. Rabbit IgG was used as a negative control. **c** AGS cells were cotransfected with Flag-c-Jun and a series of N-terminal or C-terminal HA-KDM4B mutants, followed by IP using anti-Flag. **d** KDM4B was upregulated in the presence of *H. pylori*. KDM4B was detected by anti-KDM4B antibody. β-actin was an internal control. **e** The depletion of KDM4B reduced the *IL-8* promoter activity. Cells (pLKO, sh4B#1, or sh4B#2) were cotransfected with *IL-8*-luc plus β-galactosidase in the absence (−*Hp*) or presence (+ *Hp*) of *H. pylori*. **f** The *IL-8* promoter activity of KDM4B-knockdown cells (sh4B#1 or sh4B#2) was restored by introduction of a shRNA-resistant KDM4B expression vector. KDM4B^a^ and KDM4B^b^: the #1 and #2 shRNA-resistant KDM4B vectors, respectively. **g** Introduction of the inactive KDM4B mutant, KDM4B^H189A^, displayed a lower level of the *IL-8* promoter activity than that of the wild-type KDM4B in AGS cells. The *IL-8* promoter activity was measured from AGS cells cotransfected with wild-type KDM4B (KDM4B^WT^) or inactive mutant (KDM4B^H189A^) in the absence (−*Hp*) or presence (+*Hp*) of *H. pylori*. The relative activity was normalized to that of mock vector-transfected cells. *p < 0.05; **p < 0.01

Since the expression of IL-8 is regulated by both NF-κB and AP-1^33^, we evaluated whether KDM4B cooperates with NF-κB and/or AP-1 using an IL-8 reporter plasmid in which the promoter region consists of AP-1 and NF-κB sites fused with a luciferase reporter gene (*IL-8*-luc)^49^. Depletion of KDM4B significantly attenuated the transactivation of *IL-8* promoter activity. We then compared the contribution of AP-1 or NF-κB to IL-8 reporter activity using two mutant reporter plasmids (∆AP-1 carrying a deleted AP-1-binding motif and ∆NF-κB carrying a deleted NF-κB-binding motif). The introduction of ∆AP-1 into control cells markedly reduced the transactivation activity (23% residual activity) as compared with the 83% residual activity observed using ∆NF-κB. Depletion of KDM4B significantly reduced IL-8 transactivation activity for both mutant vectors. Notably, only minor activity was detected for the KDM4B-knockdown cells transfected with ∆AP-1 (Fig. 3a). These results together suggest that KDM4B cooperates with NF-κB and AP-1 to regulate IL-8 expression by removing the repressive H3K9me3 and thereby relaxing the heterochromatin. In support of this notion, Han et al.^10^ found that KDM4B interacts with NF-κB (p65) to drive the expression of Cox2.

We thus asked whether KDM4B interacts with AP-1. Immunoprecipitation (IP) of lysates using anti-KDM4B antibodies from AGS cells showed that endogenous KDM4B interacts with c-Jun, a crucial component of AP-1. A reciprocal IP of lysates using c-Jun also detected endogenous KDM4B, suggesting that KDM4B is associated with c-Jun (Fig. 3b) and thereby interacting with AP-1. We next examined the minimal region of KDM4B crucial to interact with c-Jun using a series of N-terminal and C-terminal HA-tagged KDM4B mutants (∆N470, ∆C180, ∆C290, and ∆C460) (Fig. 3c). AGS cells were cotransfected with Flag-c-Jun plus each of the truncated HA-KDM4B clones, followed by IP and western blotting analysis. KDM4BΔC180 retained comparable association with c-Jun. KDM4BΔC290 and KDM4BΔC460 had a decreased level of association, whereas much weaker signal was detected for KDM4BΔN470 (Fig. 3c). These results revealed that *N*-terminal region of KDM4B that consists of the JmjN-JmjC-PHD region (residues 1–907) is important for interacting with c-Jun. Interestingly, infection with *H. pylori* induced an increase in KDM4B production (Fig. 3d). This increase was accompanied by an increased IL-8 reporter activity upon *H. pylori* infection. Depletion of KDM4B significantly reduced the promoter activity, irrespective of *H. pylori* infection (Fig. 3e).

We next tested whether the restoration of KDM4B expression in KDM4B-knockdown cells could rescue IL-8 promoter activity. Figure 3f shows that the introduction of a shRNA#1-resistant KDM4B expression clone, KDM4B^a^, into the sh4B#1 line significantly restored transactivation activity, without and with *H. pylori* infection. Likewise, IL-8 reporter activity for sh4B#2 could be rescued by introducing a shRNA#2-resistant KDM4B expression clone, KDM4B^b^. We also measured the release of IL-8 when KDM4B expression was restored in KDM4B-knockdown cells by using the same constructs. As seen in Figs. S4A-4B, a significantly elevated level of IL-8 production was detected in *H. pylori*-infected KDM4B-knockdown AGS cells and MKN45 cells. On the other hand, the introduction of an inactive mutant vector H189A into AGS cells significantly reduced IL-8 reporter activity as compared with that of the WT KDM4B vector without or with *H. pylori* infection (Fig. 3g). Thus, KDM4B is a crucial regulator of IL-8 transactivation via its demethylase activity. Its physical interaction with the AP-1 component c-Jun might contribute to the regulation of the IL-8 transactivation activity.

### KDM4B and c-Jun are co-recruited to the AP-1 site of the *IL-8* and *MMP1* loci

**Fig. 4:**
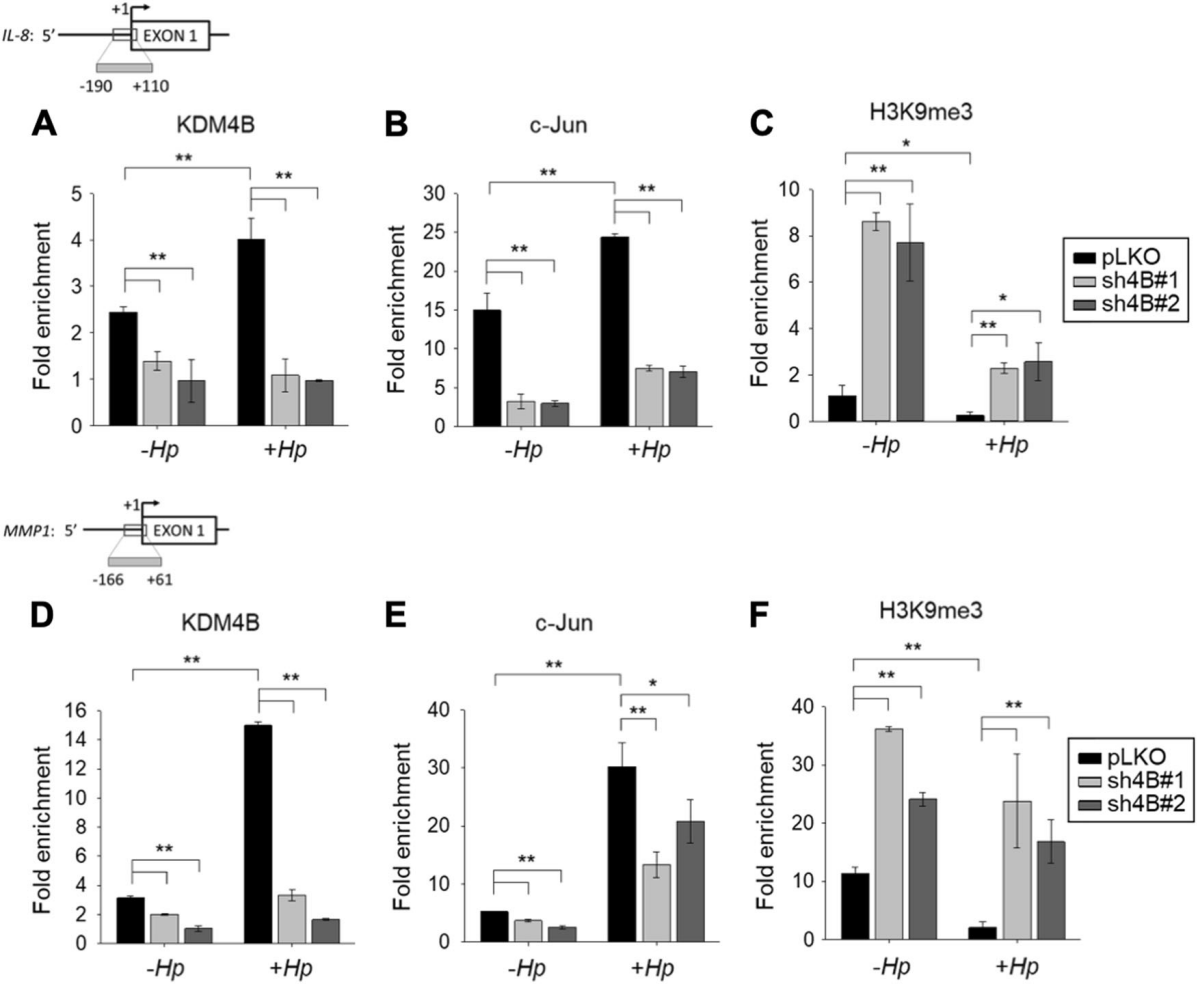
KDM4B enhances the recruitment of c-Jun to the *IL-8* and *MMP1* promoters. ChIP analysis of KDM4B and c-Jun occupancy and H3K9me3 signal on the promoter of *IL-8* (**a**‒**c**) or *MMP1* (**d**‒**f**) in non-infected AGS cells or those infected with *H. pylori*. Chromatin immunoprecipitation (ChIP) was performed with specific antibodies against KDM4B (**a**, **d**), c-Jun (**b**, **e**), and H3K9me3 (**c**, **f**), respectively. AGS cells were infected with *H. pylori* at an moi of 50 for 3 h, followed by ChIP analysis. Fold enrichments normalized to IgG are presented (mean ± SD from three independent experiments). *p < 0.05; **p < 0.01

We next determined whether KDM4B and c-Jun were co-recruited to the same target loci using a ChIP analysis with anti-KDM4B, anti-c-Jun, and anti-H3K9me3 antibodies. We showed that the depletion of KDM4B significantly reduced the occupancy of KDM4B and c-Jun (LKO vs. sh4B#1/sh4B#2) on the *IL-8* promoter region in non-infected cells (Fig. 4a, b). Infection with *H. pylori* significantly increased the occupancy of KDM4B and c-Jun. Conversely, the depletion of KDM4B (sh4B#1 or sh4B#2) essentially blocked the occupancy of c-Jun. In parallel, there was a significantly increased level of the repressive H3K9me3 mark on this locus (Fig. 4c), consistent with the reduced promoter activity of IL-8 observed in KDM4B-knockdown cells (Fig. 3e).

A similar trend was found in the ChIP analysis of the *MMP1* locus. Infection with *H. pylori* significantly increased the occupancy of KDM4B and c-Jun, and significantly decreased the H3K9me3 signal (Fig. 4d‒f). Conversely, the depletion of KDM4B led to a significantly reduced level of c-Jun occupancy (Fig. 4e), but an elevated level of H3K9me3 (Fig. 4f). These results together suggest that KDM4B affected the enrichment of c-Jun on the relevant promoters, and this effect was more pronounced in the presence of *H. pylori* challenge, hence promoting c-Jun transactivation.

### Silencing of KDM4B reduces type IV secretion system-mediated CagA signaling in *H. pylori*-infected cells

**Fig. 5:**
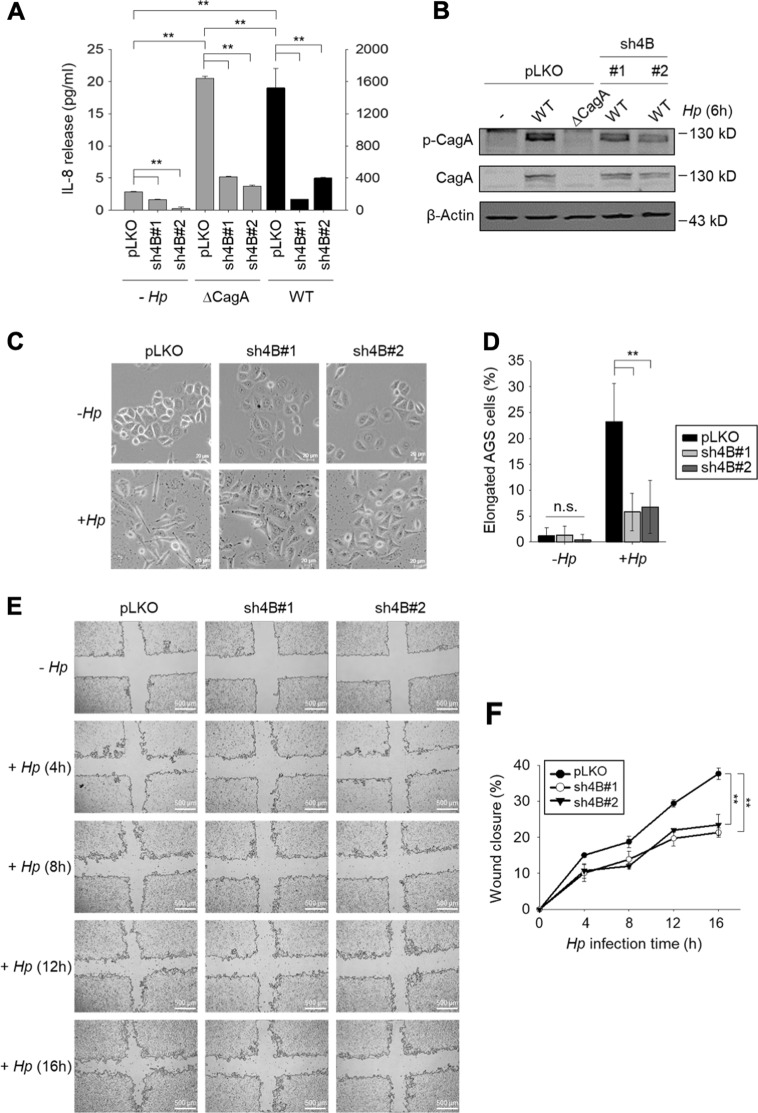
*H. pylori* CagA is essential for KDM4B-mediated IL-8 production and hummingbird phenotype formation in infected cells. **a** The CagA-positive *H. pylori*-induced IL-8 production mediated by KDM4B was significantly reduced in ∆*cagA*-infected AGS cells. Control and KDM4B-knockdown cells (sh4B#1 and sh4B#2) were either not infected or infected with WT (strain 26695) or ∆*cagA* at an moi of 50. IL-8 released into the culture supernatant was measured by ELISA. The values are means and SDs of three independent experiments. Statistical significance was evaluated using the Student’s *t*-test (*p < 0.05; **p < 0.01). **b** Detection of translocated phosphorylated CagA in KDM4B-knockdown cells was reduced. AGS cells were infected with WT or ∆*cagA H. pylori* at an moi of 50 for 6 h. Phospho-tyrosine antibody was used for determining phospho-CagA (p-CgaA) in immunoblotting assay. **c** Infection of KDM4B-knockdown cells with WT *H. pylori* had significantly reduced ability to display the hummingbird phenotype as compared with the control AGS cells. AGS cells were either not infected (−*Hp*) or infected with strain 26695 (+ *Hp*) at an moi of 50 for 3 h. Cells were visualized by phase-contrast microscopy to assess AGS cell morphology. Scale bar, 20 μm. **d** Quantification of the percentage of elongated cells from (**c**). **e** Infection of KDM4B-knockdown cells with WT *H. pylori* reduced cell migration. Wound healing effect of control (pLKO) and KDM4B-knockdown (sh4B#1 and sh4B#2) AGS cells at indicated post-infection time points. **f** The percentage of wound closure was measured from (**e**). The values are means and SDs of three independent experiments. Statistical significance was evaluated using the Student’s *t*-test. *p < 0.05; **p < 0.01

The key virulence factor CagA can be translocated and become phosphorylated, inducing aberrant cell signaling and the substantial upregulation of IL-8^38,40^. We asked whether KDM4B was involved in CagA-mediated IL-8 production. As shown in Fig. 5a, IL-8 production was significantly lower in KDM4B-knockdown cells infected with either WT or ∆*cagA* (Fig. 5a). Significantly upregulated production of IL-8 was only seen in control cells infected with WT *H. pylori*. We next compared the levels of translocated/phosphorylated CagA in control (pLKO) vs. KDM4B-knockdown cells infected with *H. pylori*. Infection of KDM4B-knockdown cells with WT *H. pylori* greatly reduced the levels of phosphorylated CagA as compared with those of uninfected control cells, suggesting that KDM4B is crucial for the translocation of CagA into infected cells (Fig. 5b).

We also evaluated the formation of an elongated cell, referred to as a hummingbird phenotype that is p-CagA-dependent in cells without or with *H. pylori* infection^50,51^. Figure 5c, d shows that infected KDM4B-knockdown cells displayed a significantly reduced level of the hummingbird phenotype (*p* < 0.01). Additionally, we determined whether the knockdown of KDM4B affected cell migration mediated by translocated CagA using a wound healing assay^52^. Infected KDM4B-depleted cells also exhibited a significantly reduced wound healing effect at 16-h post-infection (Fig. 5e, f). Thus, these results together substantiate the notion that KDM4B regulates the translocation of CagA upon *H. pylori* infection.

### KDM4B directly regulates the expression of *ITGAV* encoding integrin αV to facilitate the translocation of CagA

**Fig. 6:**
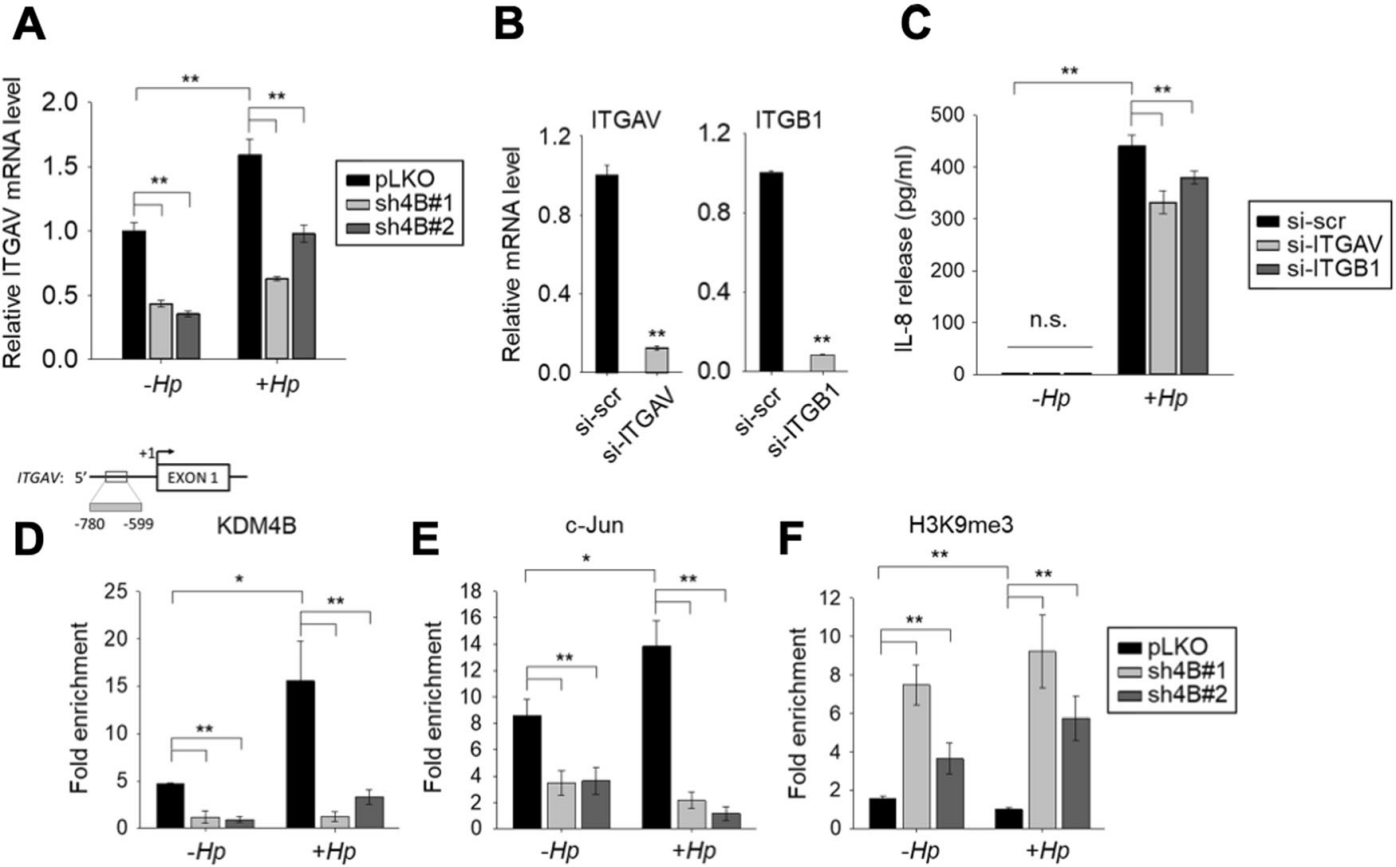
KDM4B regulates the expression of integrin αV. **a** Reduced expression of integrin αV encoded by ITGAV in KDM4B-knockdown cells. qRT-PCR analysis was performed to evaluate the expression of ITGAV in pLKO, sh4B#1, and sh4B#2 cells, respectively. Cells were either not infected (−*Hp)* or infected with strain 26695 (+ *Hp*) at an moi of 50 for 3 h. **b**, **c** Silencing of integrin αV and β1 reduces *H. pylori*-stimulated IL-8 production. AGS cells were transfected with control (si-scr), integrin αV (si-ITGAV), or β1 (si-ITGB1) siRNA and cultured for 24 h, followed by non-infection (−*Hp*) or infection with *H. pylori* (+ *Hp*) at an moi of 50 for 3 h. Total RNAs were prepared and subjected to qRT-PCR for ITGAV and ITGB1 expression (**b**). IL-8 released into the culture supernatant was measured by ELISA (**c**). **d**–**f** KDM4B is recruited at the *ITGAV* locus. Chromatin was prepared from pLKO, sh4B#1, or sh4B#2 cells with anti-KDM4B (**d**), c-Jun (**e**), or H3K9me3 (**f**), followed by quantitative PCR analysis. Fold enrichments normalized to IgG are presented. The values are means and SDs of three independent experiments. Statistical significance was evaluated using the Student’s *t*-test. *p < 0.05; **p < 0.01

We reasoned that the integrin protein responsible for interactions with CagL (the RGD-containing protein) on the pilus of the type IV secretion system to deliver CagA^53^ was regulated by KDM4B. Microarray data showed no significant differential expression of genes encoding integrin α5 and β1^53^. Instead, another integrin member, *ITGAV*, encoding integrin αV was significantly downregulated in the two KDM4B-knockdown lines (sh4B#1 and sh4B#2) as compared with control cells (Fig. 6a). *H. pylori*-infected cells exhibited a higher level of *ITGAV* expression than that of non-infected cells, indicating that KDM4B regulated the expression of *ITGAV*.

Integrin αV is highly expressed in AGS cells^53^ and interacts with integrin β1 to form a heterodimer that binds to RGD-containing proteins^54^. We tested whether αVβ1 mediates IL-8 production in *H. pylori*-infected cells. AGS cells treated with si-scr treatment, when non-infected, had a low signal of IL-8 production (Fig. 6c) as compared with a slightly higher level of IL-8 production in non-infected pLKO cells (Fig. 1b). We speculate that the variation of IL-8 release stemmed from different approaches utilized; while the pLKO control was a stable line derived based on the delivery of pLKO-shRNA, si-scr control was conducted by introduction of AGS with si-scr under a transient transfection condition. Despite slight difference in the level of IL-8, they represent a relatively small value (<5%) as compared with those in infected cells. Depletion of *ITGAV* by siRNA (si-ITGAV), interestingly, led to a significantly lower level of IL-8 production in cells infected with *H. pylori* as compared with scramble siRNA (si-scr)-treated cells (Fig. 6b, c). A comparable result was also obtained for cells depleted of integrin β1 (si-*ITGB1*). Similar effects were also found for MKN45 cells (Figure S3C), together suggesting that the expression of *IL-8* and *ITGAV* was regulated by KDM4B and could be stimulated by *H. pylori* challenge.

We further asked whether KDM4B and c-Jun were co-recruited to the promoter region of *ITGAV* using a ChIP analysis. Depletion of KDM4B (sh4B#1 and sh4B#2) significantly reduced the occupancy of KDM4B and c-Jun at the *ITGAV* locus (Fig. 6d, e). Infection with *H. pylori* significantly increased the recruitment of both KDM4B (*p* < 0.01) and c-Jun (*p* < 0.05) as compared with that in non-infected control cells. Conversely, a significantly reduced level of KDM4B and c-Jun on the *ITGAV* locus (*p* < 0.01) was seen for infected KDM4B-knockdown cells as compared with the infected control cells. Furthermore, there was a significant increase of the repressive H3K9me3 mark at this locus (Fig. 6f), indicating that KDM4B contributes to demethylation activity. Taken together, these results suggest that KDM4B and c-Jun regulate the expression of *ITGAV*, which encodes integrin αV, to facilitate the translocation of CagA upon *H. pylori* infection, at least in part.

### KDM4B expression and p-c-Jun abundance are positively correlated in gastric cancer

**Fig. 7:**
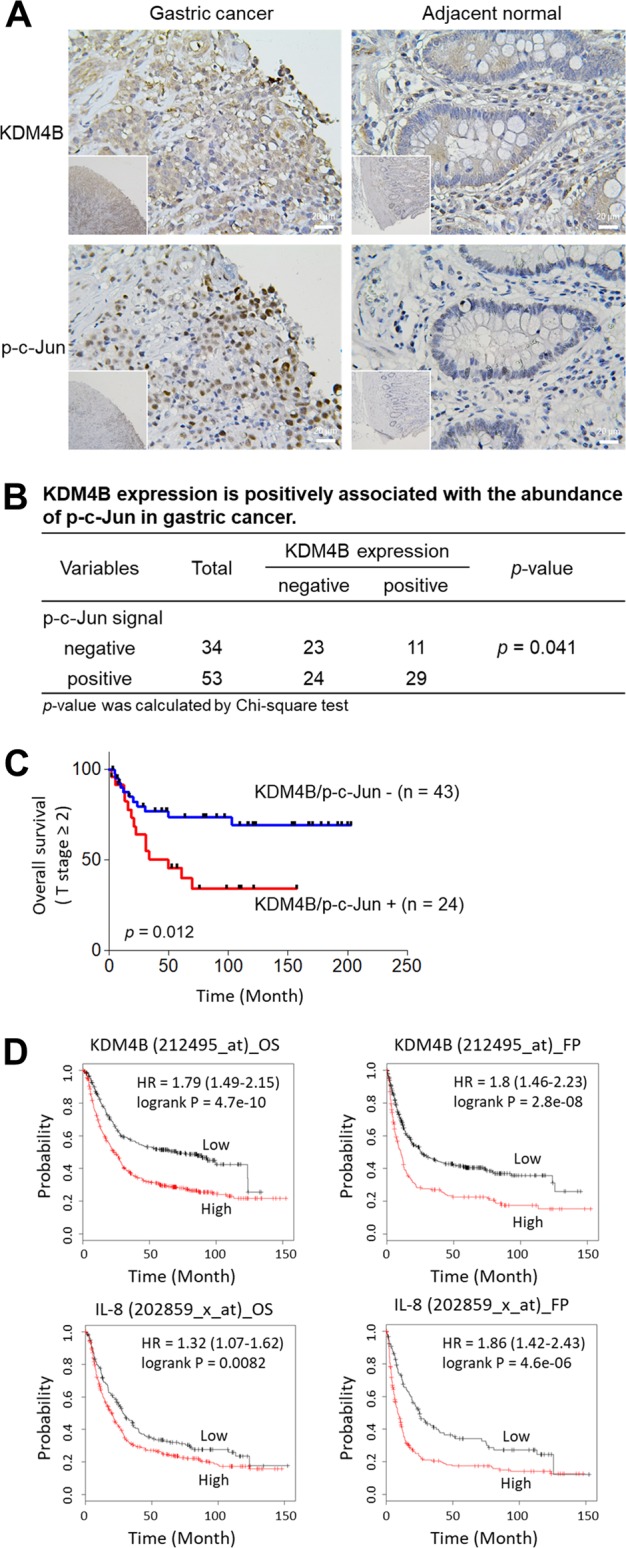
Elevated expression of KDM4B and the abundance of p-c-Jun are clinically associated in gastric cancer. **a** Immunohistochemical detection of KDM4B and p-c-Jun in gastric tissue specimens. The expression level of KDM4B and the signal of p-c-Jun in gastric tissue specimens are elevated in the cancerous site as compared with the adjacent normal site. **b** The correlation between KDM4B expression and p-c-Jun signal in 87 gastric cancer biopsies. Statistical significance was evaluated using the Chi-square test. **c** Overall survival of GC patients with co-expression of KDM4B and p-c-Jun in advanced gastric cancer biopsies defined by the Kaplan–Meier curves. Overall survival of patients with advanced T stage (T2−T4; *n* = 67) were assessed. +, positive expression in both KDM4B and p-c-Jun; –, negative expression in one of KDM4B and p-c-Jun or none. **d** Kaplan–Meier survival curves. KM plots of gastric cancer survival for patients with KDM4B and IL-8 expression

KDM4B overexpression is associated with tumor growth in gastric cancer^55^. To further determine the clinical relevance of KDM4B and p-c-Jun in gastric cancer, we examined the expression of KDM4B and the abundance of p-c-Jun in two consecutive sections of paraffin-embedded gastric cancer specimens (total, *n* = 87; intestinal, *n* = 18; diffuse-type, *n* = 28; mix, *n* = 11; unknown, *n* = 30) by immunohistochemistry. Forty out of 87 showed positive KDM4B staining in tumor tissue nuclei, but no signal in normal tumor-adjacent regions (Fig. 7a). The nuclear signal of p-c-Jun was clearly visible in the tumor region of 53 subjects in the cancerous region, and no signal was observed in the normal tumor-adjacent region. The expression of KDM4B was significantly positively correlated with the abundance of p-c-Jun (*p* = 0.041, Fig. 7b). We then evaluated the clinical relevance of co-expression of both KDM4B and p-c-Jun using Kaplan–Meier analysis. Strikingly, the co-expressed KDM4B-p-c-Jun group was significantly associated with a low overall survival rate among patients with advanced T stage (T2–T4; *n* = 67) as compared with only one or no expression of both markers (Fig. 7c).

We next used the Kaplan–Meier (KM) approach (KM Plotter: http://kmplot.com) to analyze gene expression in patients with gastric cancer with respect to the endpoints of overall survival (OS, *n* = 876) and first progression (FP, *n* = 641)^43^. A KM plotter analysis revealed that elevated expression of KDM4B was significantly associated with worse OS and FP outcomes (Affy ID: 212495_at, 212492_s_at, 215616_s_at, 216023_at) (Fig. 7d and Table S1). When patients were classified into cohorts based on Lauren histological subtypes, both the intestinal and diffuse types carrying elevated expression of KDM4B had worse OS and FP. Furthermore, high expression of IL-8 was associated with a worse clinical outcome in all-type gastric cancer as well as in intestinal- and in diffuse-type gastric cancer (Fig. 7d and Table S2). These results suggest that elevated KDM4B expression is significantly associated with the abundance of p-c-Jun, which is a major stress-activated protein, thus regulating the IL-8 expression in gastric cancer and being linked to a poor clinical outcome.

## Discussion

KDM4A and KDM4B are considered promising therapeutic targets for prostate and breast tumor growth based on their functions as coactivators of androgen receptor and estrogen receptor, respectively^1,5,23^. KDM4A recruits E2F1 to control cancer metabolism for proficient prostate cancer growth^14^. Here, we show that the silencing of KDM4B, but not KDM4A/KDM4C, with specific shRNAs significantly reduced IL-8 production in gastric cancer cells. This effect was significantly triggered in cells stressed by *H. pylori* infection, indicating an exclusive pathogenic function. IL-8 is well documented for its ability to activate inflammation-mediated processes and angiogenesis in a variety of cancers, including prostate and gastric cancers^56,57^. Elevated expression of IL-8 also induces proliferation and migration and is correlated with a poor prognosis in gastric cancer^58^. In this study, we provide evidence that KDM4B functions as a novel coactivator of c-Jun to mediate AP-1-controlled genes, including *IL-8* and *MMP1*, based on several lines of evidence: (i) Microarray data show the significant differential expression (i.e., downregulation) of c-Jun targets in response to stimuli between LKO control and KDM4B-knockdown cells. (ii) KDM4B physically interacts with c-Jun. (iii) KDM4B and c-Jun are co-recruited to *IL-8*, *MMP1*, and *ITGAV* promoters. (iv) The depletion of KDM4B significantly reduces the IL-8 production and occupancy of *c-Jun*, but increases H3K9me3 at the promoter region of *IL-8* and *MMP1*. Although KDM4A and KDM4B share a highly conserved JmjC domain and similar primary-structure organization, their C-terminal PHD and Tudor domains are less homologous (sequence identity, ~50%)^2,3^. Recent evidence shows that they display an overlapping, but distinct spatiotemporal expression profile, allowing them to function individually and combinatorially to control specific subsets of gene targets^24,25^. Notably, KDM4B but not KDM4A is a hypoxia-inducible epigenetic factor^59,60^, despite that they often exhibit elevated expression in gastric cancer (Figure S5). Furthermore, KDM4B is induced by *H. pylori* challenge, but inhibited by a JNK inhibitor, suggesting that it has a unique role under stress conditions. Taken together, our results suggest that KDM4B recruits c-Jun and promotes IL-8 production via JNK/c-Jun signaling, possibly involving a positive feed-forward loop under stress-associated conditions in gastric cancer (Figure S6).

We demonstrate that KDM4B and c-Jun regulate CagA-dependent signaling via integrin αV, a RGD-binding receptor^61^, upon *H. pylori* infection. Previously, the initial contact point between *H. pylori* and epithelial cells has been identified as the bacterial pilus adhesion molecule CagL carrying the RGD motif and the host integrin α5β1, which leads to subsequent CagA delivery by the type IV secretion system to propagate the CagA cascade, activating AP-1 (c-Jun and c-Fos) and inducing IL-8 secretion^45,53,62^. We show that integrin αV could also contribute to this effect, at least in part. First, the knockdown of KDM4B resulted in decreased *ITGAV* expression and a reduced occupancy of KDM4B on the *ITGAV* promoter. Second, CagA phosphorylation was significantly reduced in KDM4B-knockdown-infected cells as compared with infected control cells. Third, knocking down the expression of *ITGAV* significantly reduced the IL-8 production induced by *H. pylori*. Interestingly, infection with *H. pylori* induced the expression of both KDM4B and *ITGAV*, thereby augmenting the propagation of CagA signaling, phosphorylation of c-Jun, and activation of IL-8 expression via the KDM4B-c-Jun complex upon *H. pylori* infection.

KDM4B is overexpressed in gastric tissues, and plays an oncogenic role in gastric carcinogenesis^56^. Of note, the tumor suppressor miR-491-5p shows a reduced expression in gastric tissues and blocks cell proliferation and survival^55,63^. KDM4B interacts with NF-κB (p65) and regulates the expression of Cox-2, an isoform of cyclooxygenase involved in angiogenesis, in *H. pylori*-infected AGS cells^10^. We show that the infection of KDM4B-knockdown cells led to the reduced production of IL-8 as well as CXCL1 and CCL5, which are chemokines involved in gastric cancer progression^64,65^. We further demonstrate that KDM4B recruited c-Jun for efficient transcriptional activation of *IL-8* in a demethylase-dependent manner. While *H. pylori*-infected cells triggered both ERK and JNK signaling, which can stimulate IL-8 production, deletion of the AP-1 site drastically reduced IL-8 transactivation activity as compared with that observed for the deletion of the NF-κB-binding motif in KDM4B-knockdown infected cells. Immunohistochemistry analysis of gastric cancer biopsy specimens revealed that elevated KDM4B expression was significantly associated with the abundance of p-c-Jun in cancerous tissues. Notably, those advanced T-stage patients bearing positive expression of both KDM4B and p-c-Jun harbor a worse survival rate, suggesting co-expression of KDM4B and p-c-Jun as a potential prognostic marker. Furthermore, the overexpressions of *KDM4B* and *IL-8* are linked to a poor overall survival. Of note, KDM4B is associated with chromosomal instability in breast cancer; the overexpression of exogenous KDM4B leads to the loss of centromere-associated H3K9me3 and elevated chromosomal instability^66^. Although the initiation and progression of such a functional KDM4B-c-Jun loop in gastric carcinogenesis requires more elaborate mechanistic elucidation, we propose that KDM4B, an inducible epigenetic factor under stress conditions, contributes to the deregulation of the paracrine/autocrine gastric microenvironment and angiogenesis. Targeting KDM4B, a coactivator of c-Jun and NF-κB, is a potential new strategy for effective therapeutic intervention in gastric cancer progression.

## Supplementary information

Figure S1

Figure S2

Figure S3

Figure S4

Figure S5

Figure S6

Table S1

Table S2

Table S3

Table S4

Supplementary figure legends
