# Supplementary figures and images for: KDM4B is a coactivator of c-Jun and involved in gastric carcinogenesis
Source: Cell Death Dis. 2019 Jan 25;10(2):68. doi: 10.1038/s41419-019-1305-y (PMC6347645; doi:10.1038/s41419-019-1305-y)

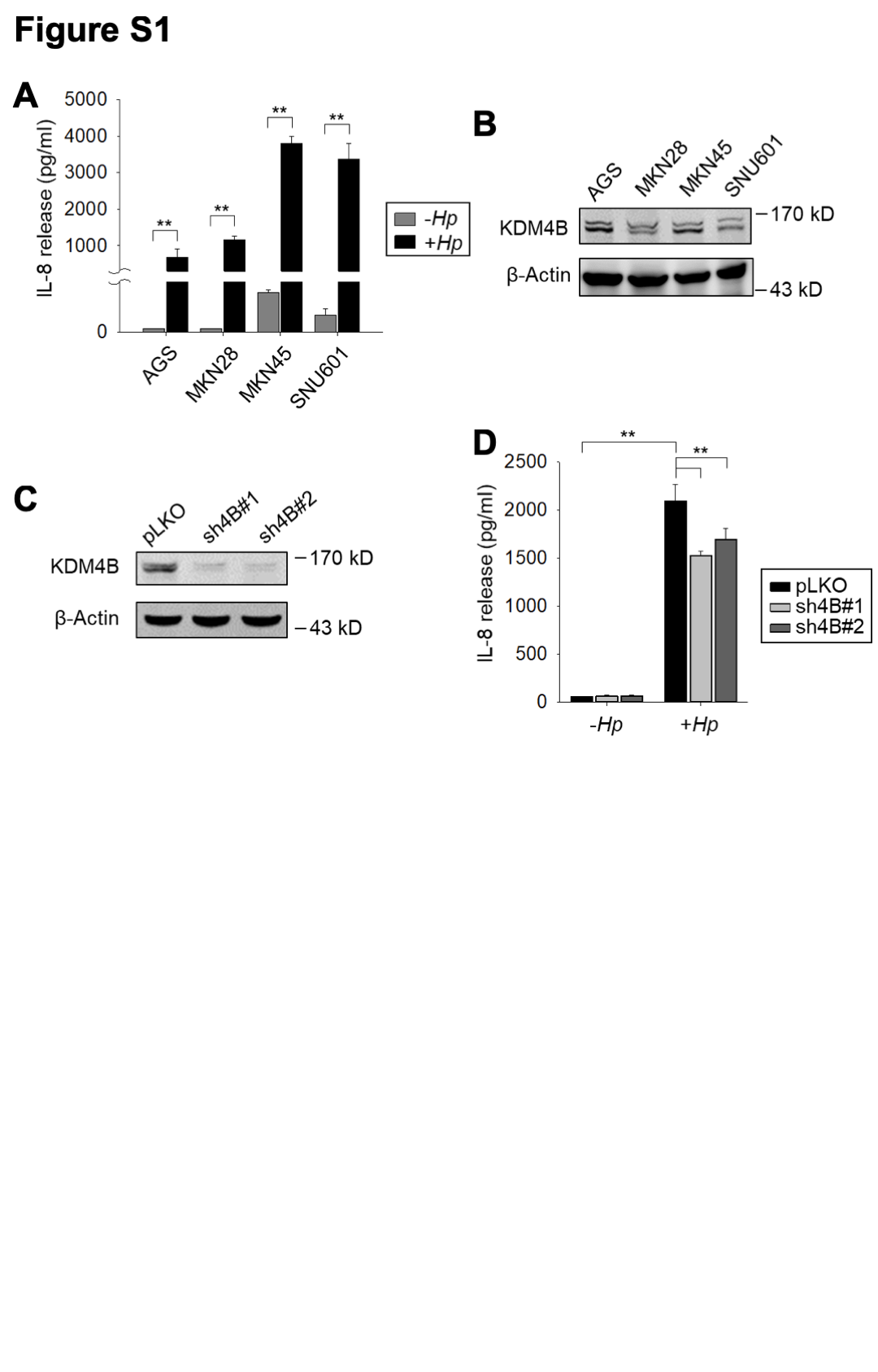

Supplement: Supplementary file 1 — Figure S1 [file 41419_2019_1305_MOESM1_ESM.tif]

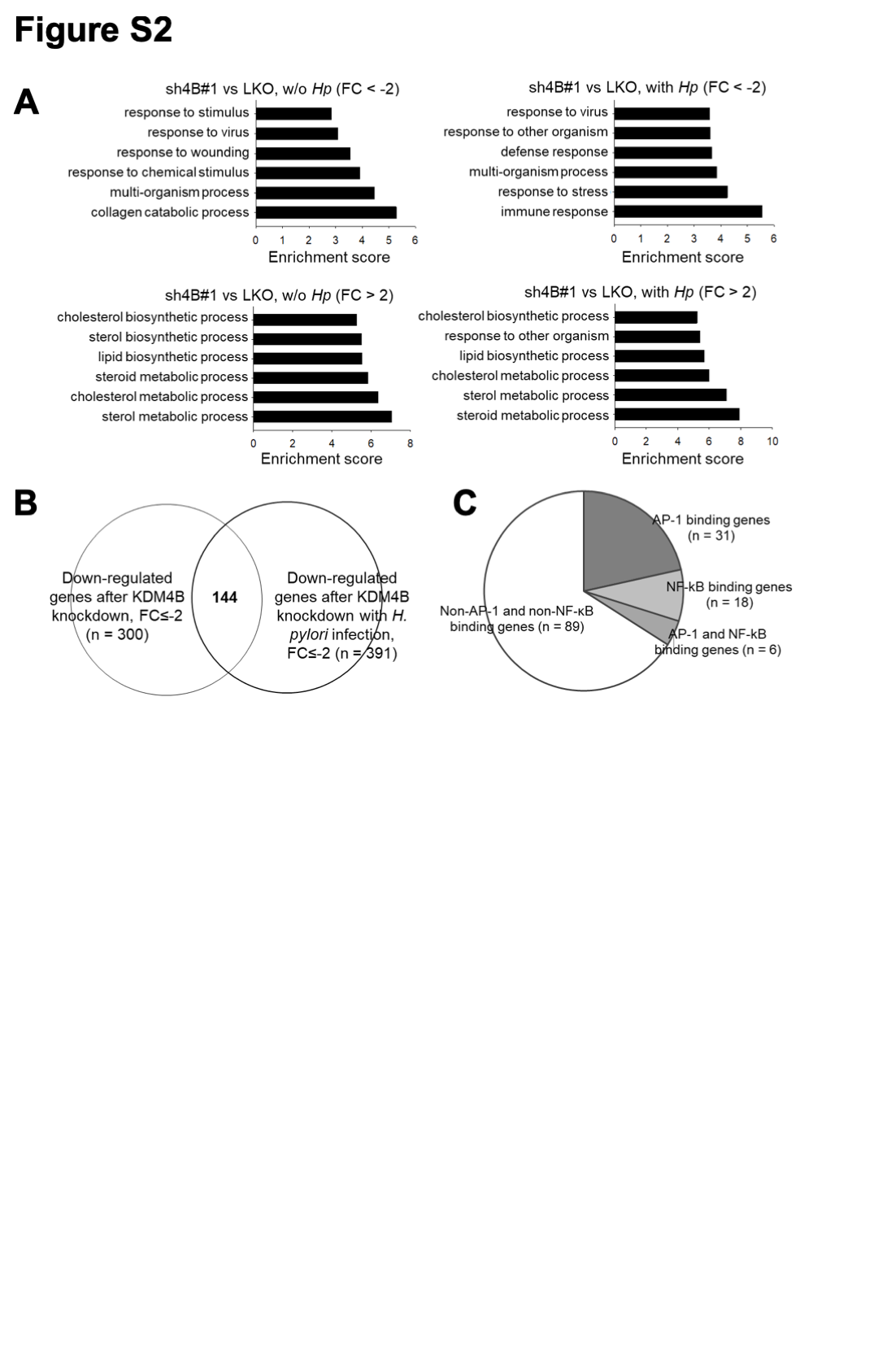

Supplement: Supplementary file 2 — Figure S2 [file 41419_2019_1305_MOESM2_ESM.tif]

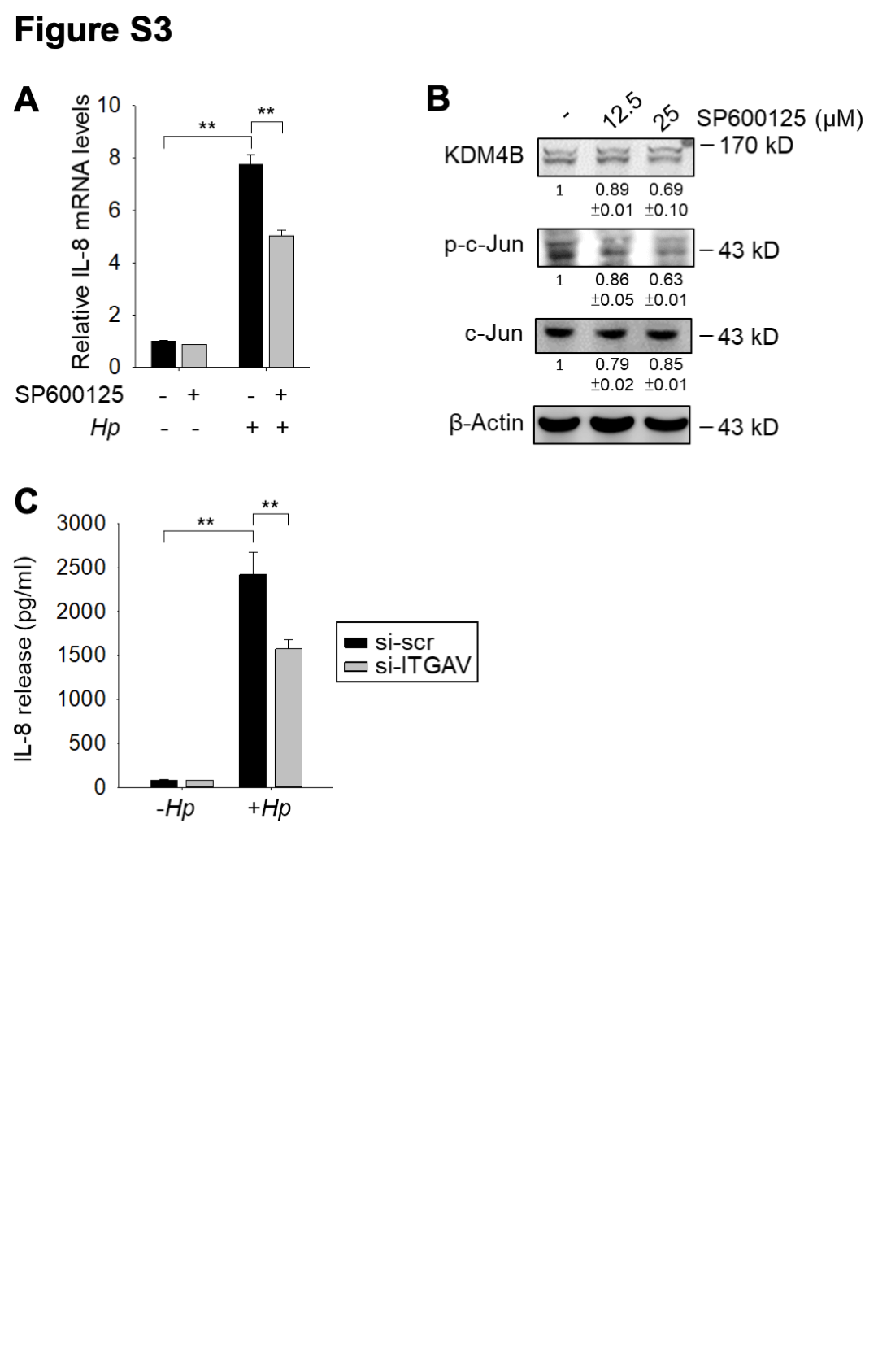

Supplement: Supplementary file 3 — Figure S3 [file 41419_2019_1305_MOESM3_ESM.tif]

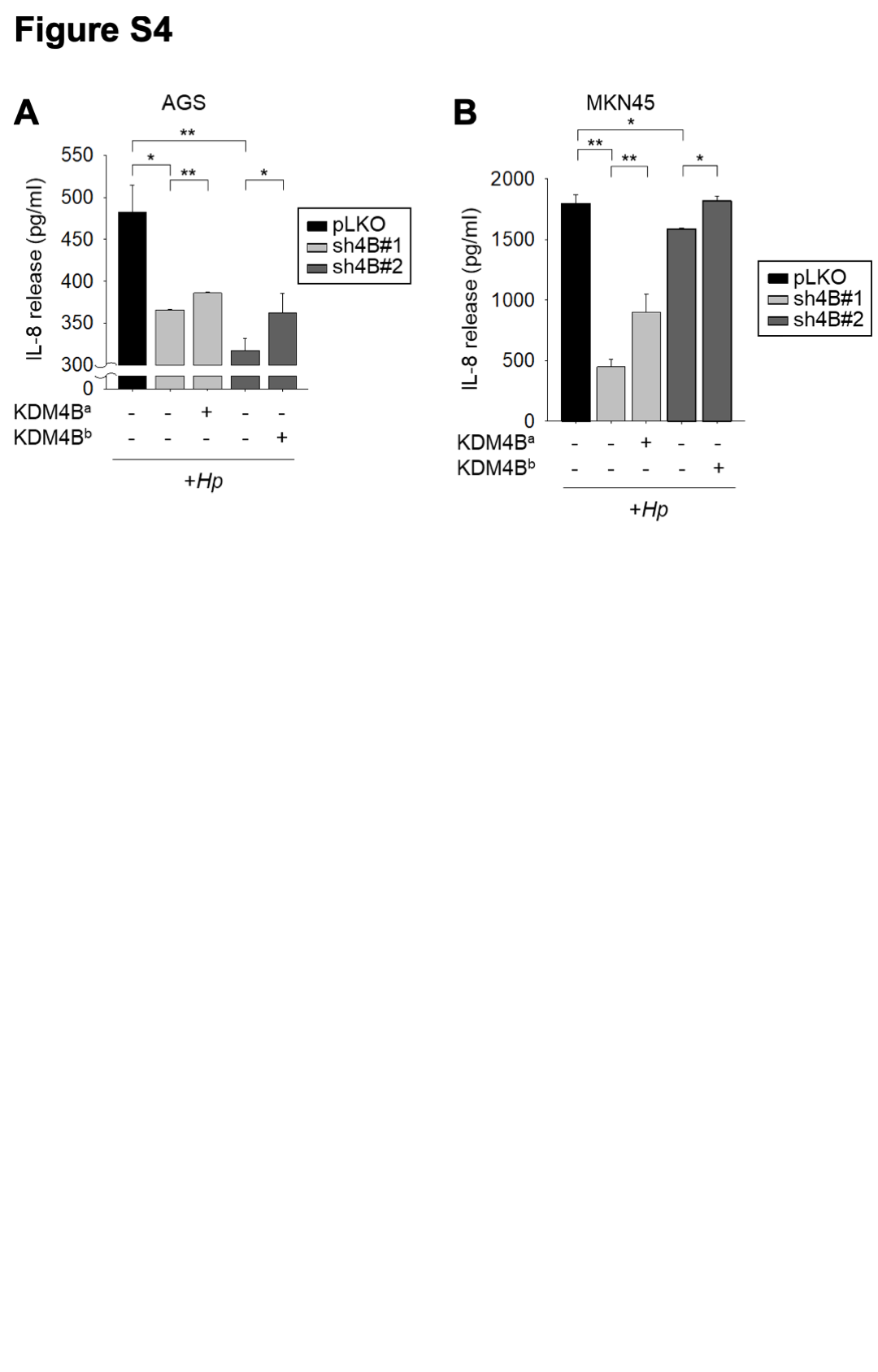

Supplement: Supplementary file 4 — Figure S4 [file 41419_2019_1305_MOESM4_ESM.tif]

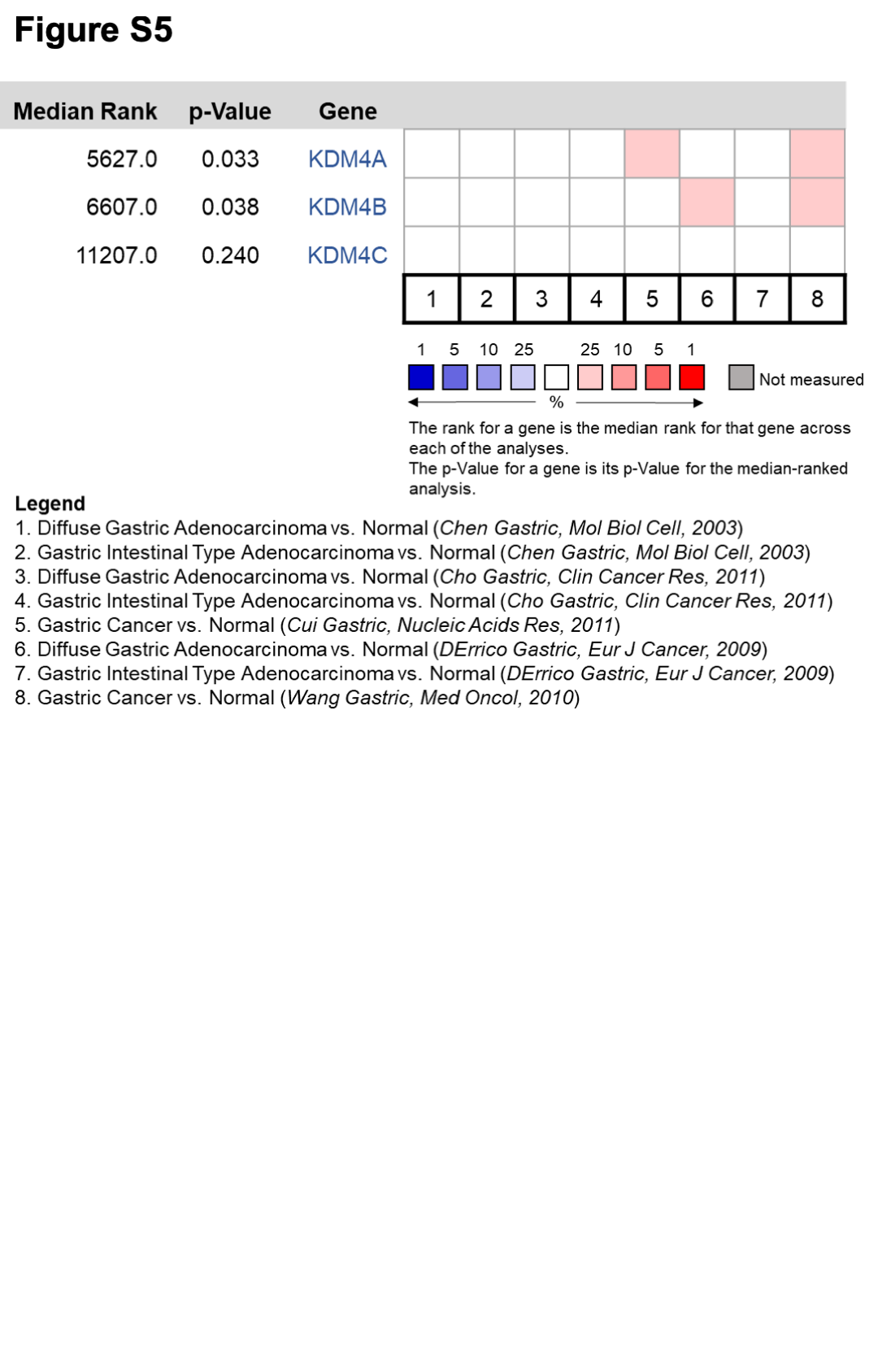

Supplement: Supplementary file 5 — Figure S5 [file 41419_2019_1305_MOESM5_ESM.tif]

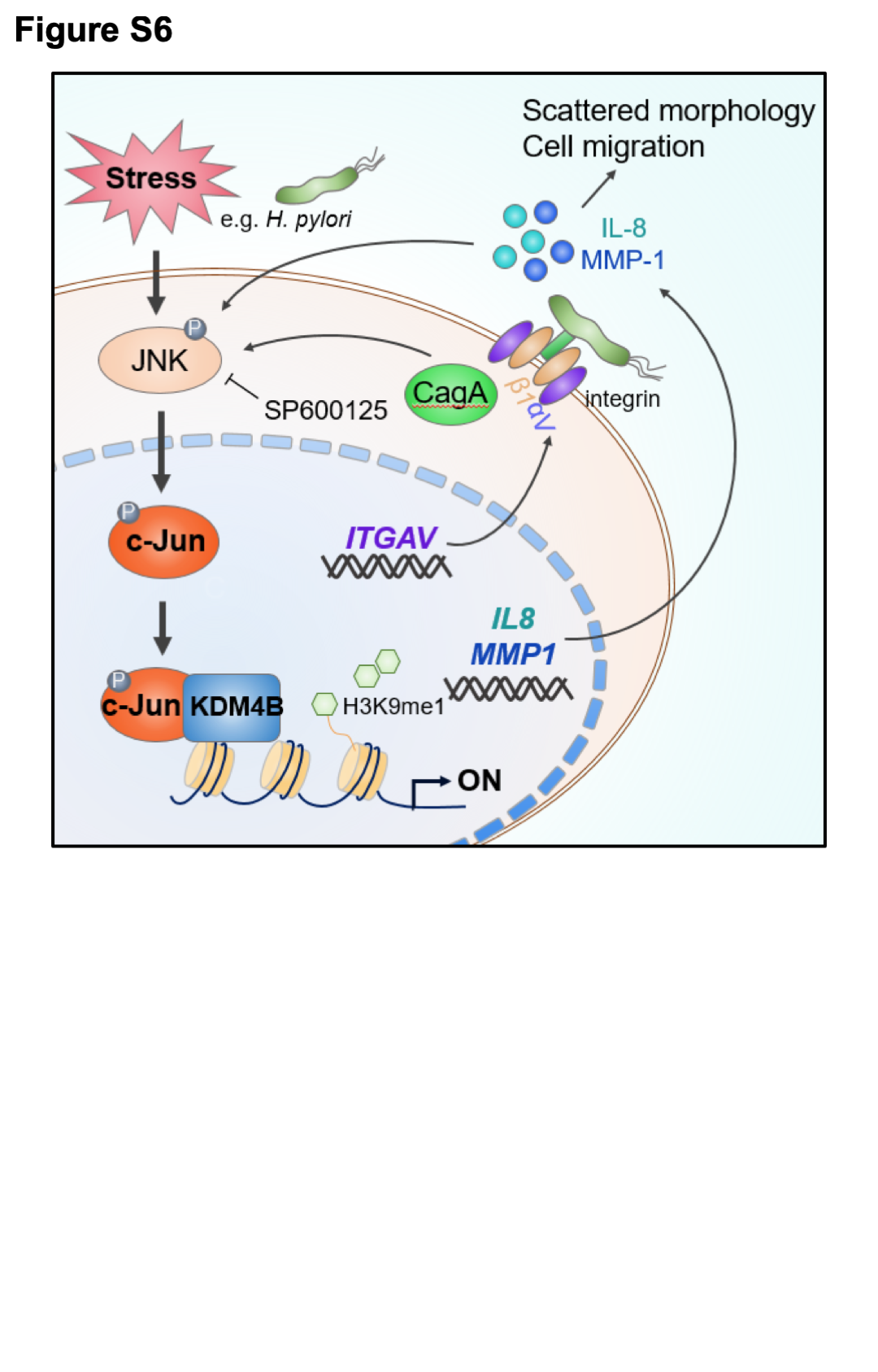

Supplement: Supplementary file 6 — Figure S6 [file 41419_2019_1305_MOESM6_ESM.tif]

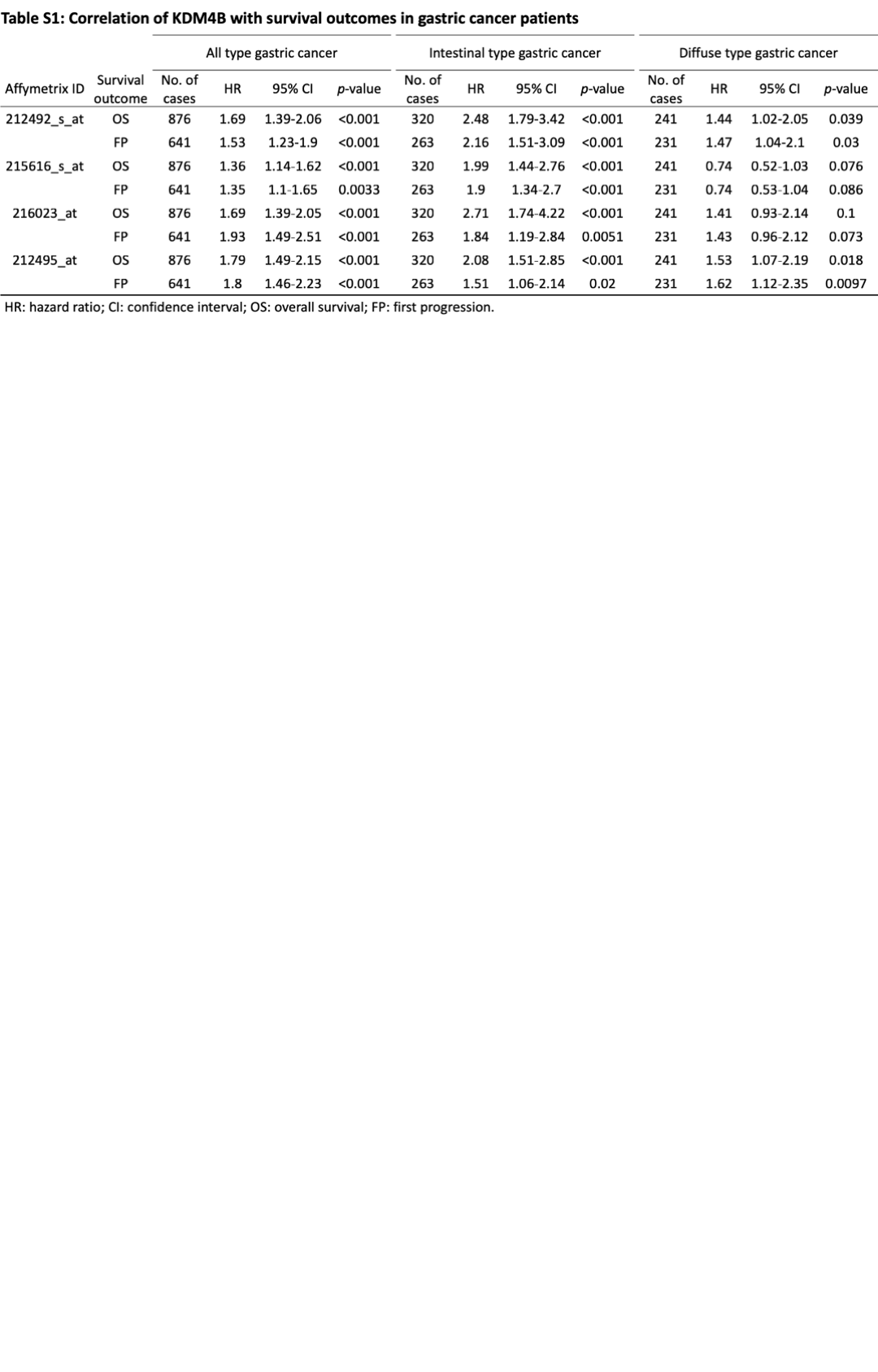

Supplement: Supplementary file 7 — Table S1 [file 41419_2019_1305_MOESM7_ESM.tif]

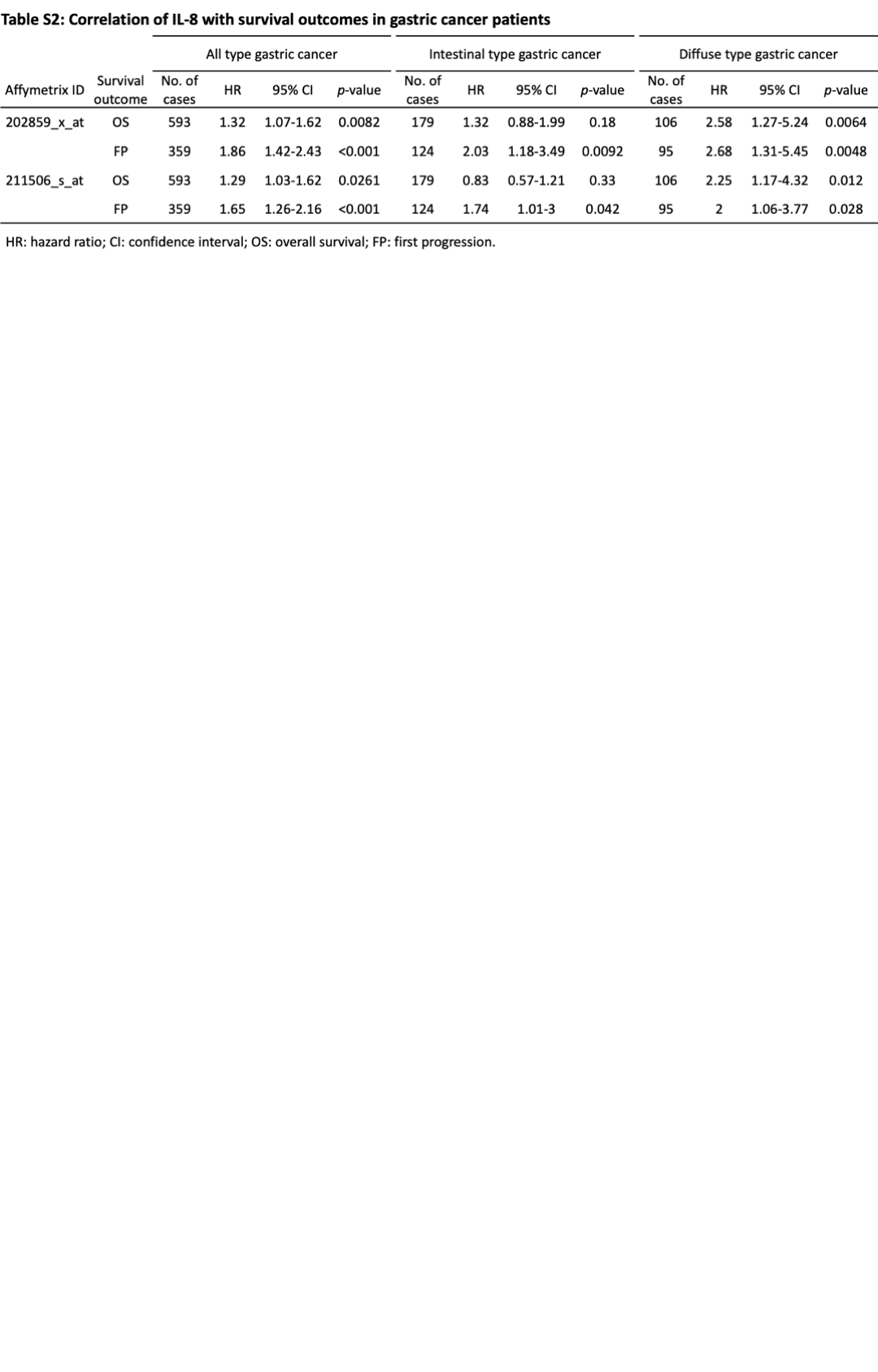

Supplement: Supplementary file 8 — Table S2 [file 41419_2019_1305_MOESM8_ESM.tif]

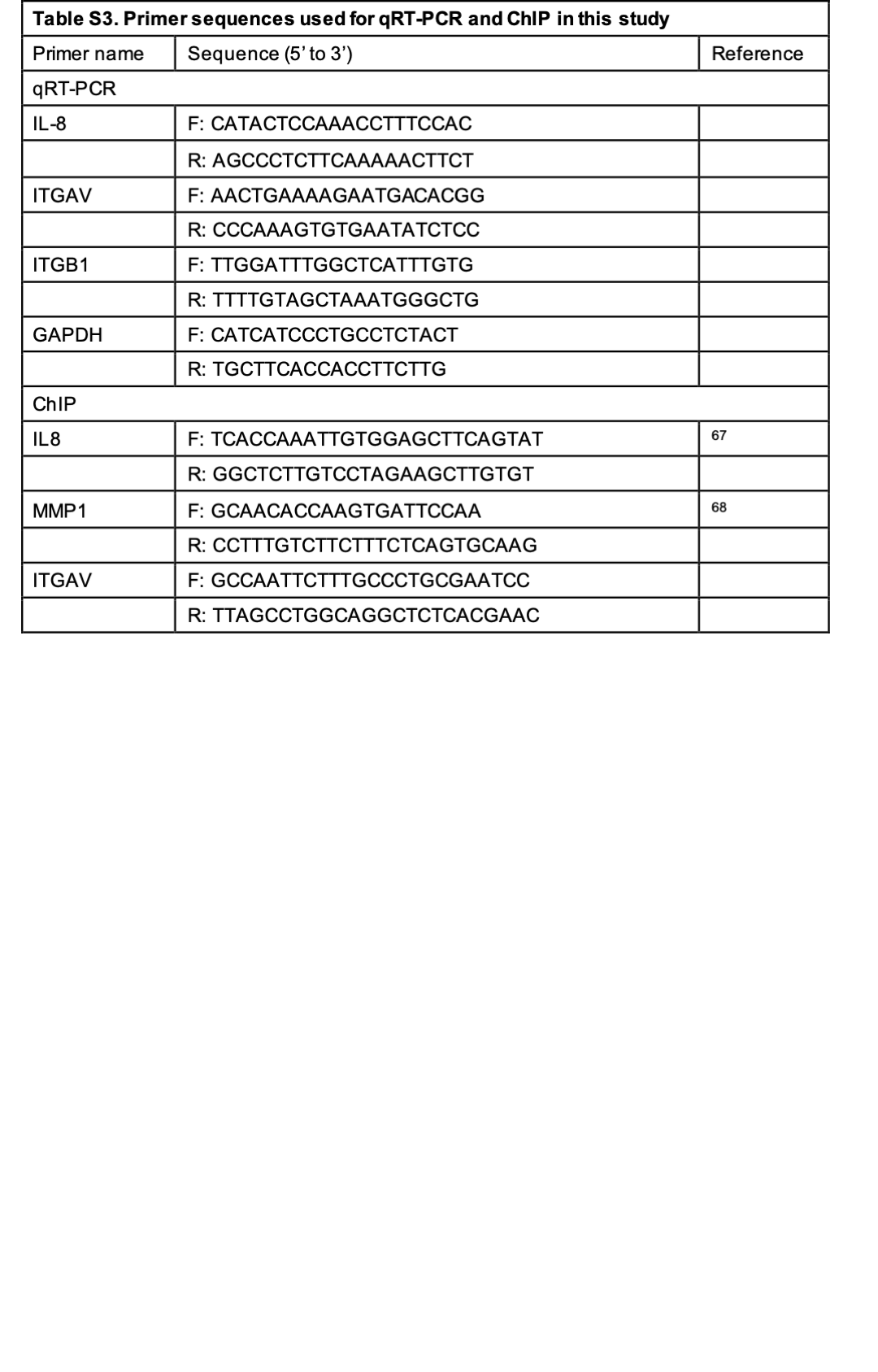

Supplement: Supplementary file 9 — Table S3 [file 41419_2019_1305_MOESM9_ESM.tif]

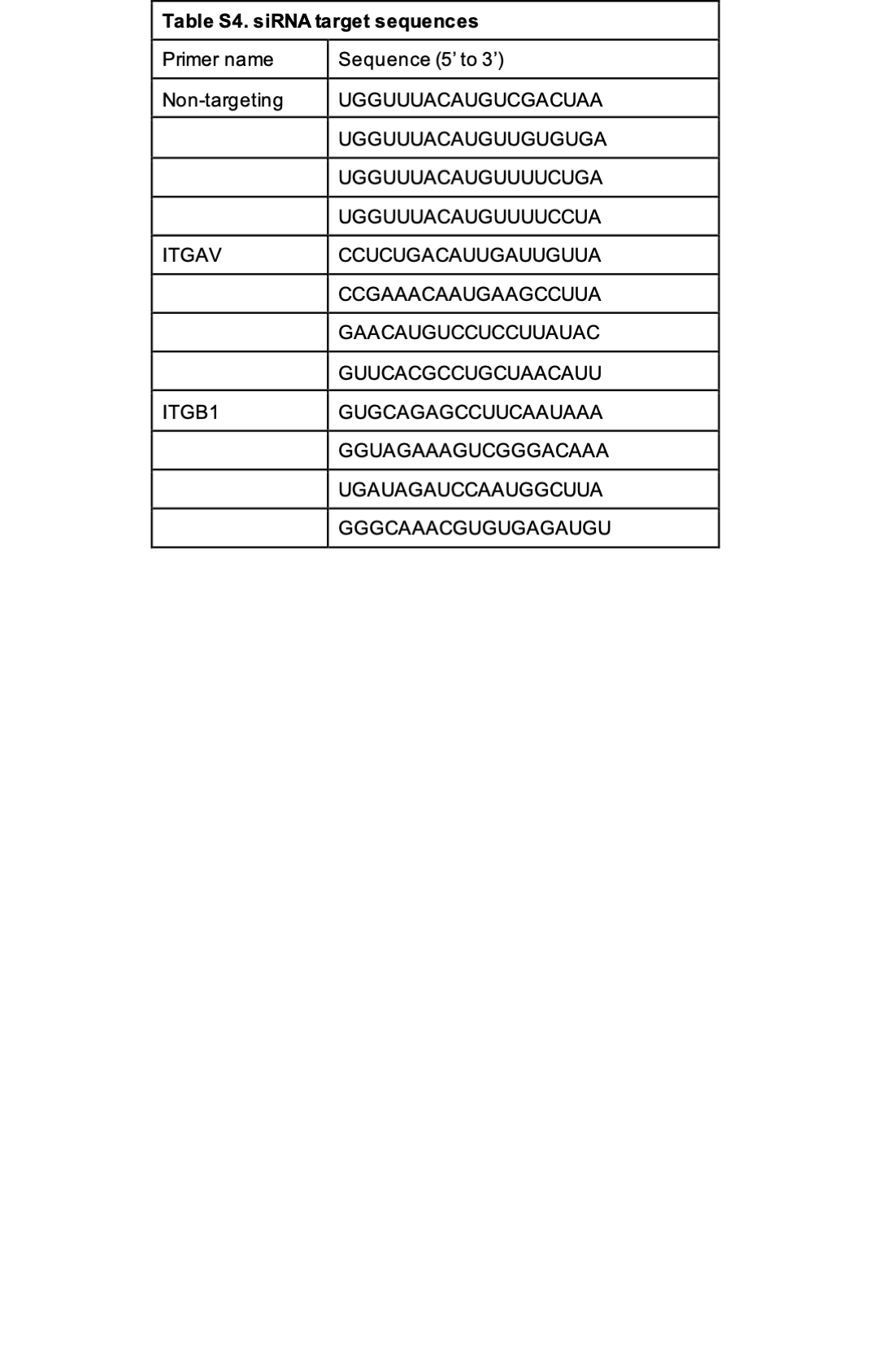

Supplement: Supplementary file 10 — Table S4 [file 41419_2019_1305_MOESM10_ESM.tif]
