# Supplementary material for: KDM4B is a coactivator of c-Jun and involved in gastric carcinogenesis
Source: Cell Death Dis. 2019 Jan 25;10(2):68. doi: 10.1038/s41419-019-1305-y (PMC6347645; doi:10.1038/s41419-019-1305-y)
Supplement: Supplementary file 11 — Supplementary figure legends [file 41419_2019_1305_MOESM11_ESM.docx]

**CDDIS-18-1334R**

**KDM4B is a co-activator of c-Jun and involved in gastric carcinogenesis**

**Supplementary figure legend**

**Figure S1.** KDM4B regulates the level of IL-8 in MKN45 cells. (A) The expression of KDM4B in AGS, MKN28, MKN45, and SNU601 gastric cancer cell lines were detected by western blotting analysis. (B) IL-8 release from the supernatants of non-infected (-*Hp*) or *H. pylori*-infected (+*Hp*) various wild-type gastric cancer cells was measured by ELISA. (C) Generation of KDM4B-control (pLKO) and KDM4B-knockdown (sh4B#1 and sh4B#2) MKN45 cells. Depletion of KDM4B was confirmed by western blotting analysis. β-Actin was the internal control. (D) IL-8 release from the culture supernatants of non-infected (-*Hp*) or *H. pylori*-infected (+*Hp*) KDM4B-knockdown MKN45 cells was measured by ELISA. (E, F) The IL-8 production was rescued by introduction of a shRNA-resistant KDM4B expression vector in AGS (E) and MKN45 (F) cells. KDM4B^a^ and KDM4B^b^: the #1 and #2 shRNA-resistant KDM4B vectors, respectively. Data represent the mean ± standard deviation (SD) from 3 independent experiments. Statistical significance was evaluated using the student’s t-test. *, *p* < 0.05; **, *p* < 0.01.

**Figure S2.** KDM4B regulates the expression of NF-κB and AP-1 target genes. (A) The Gene Ontologies (GO) biological processes of KDM4B-knockdown (sh4B#1) cells gene expression in 2-fold alterations in microarray analysis were analyzed from DAVID functional annotation tool. The bars indicate the enrichment score (-log(*p*-value)). (B) Overlapped genes between 2-fold down-regulated genes from uninfected and *H. pylori*-infected KDM4B-knockdown cells (GEO: [GSM2876232](https://www.ncbi.nlm.nih.gov/geo/query/acc.cgi?acc=GSM2876232) and [GSM287623](https://www.ncbi.nlm.nih.gov/geo/query/acc.cgi?acc=GSM2876232)3) in microarray analysis. (C) Analysis of AP-1 and NF-κB binding genes in overlapped genes from (B) by UCSC genome browser.

**Figure S3.** c-Jun and *ITGAV* regulate IL-8 production in MKN45 cells. (A) IL-8 mRNA levels were measured from MKN45 cells untreated or treated with SP600125 (25 μM) in the absence (-*Hp*) or presence of *H. pylori* (+*Hp*) at an moi of 50 for 3 h. (B) The abundance of KDM4B, p-c-Jun, c-Jun and β-actin in SP600125-treated MKN45 cells was detected by immunoblotting. (C) Silencing of integrin αV reduces *H. pylori*-stimulated IL-8 production. MKN45 cells transfected with control (si-scr) or integrin αV (si-ITGAV) siRNA and cultured for 24 hours, followed by non-infection (-*Hp*) or infection with *H. pylori* (+*Hp*) at an moi of 50 for 3 h. IL-8 released into the culture supernatant was measured by ELISA. Data represent the mean ± standard deviation (SD) from 3 independent experiments. Statistical significance was evaluated using the student’s t-test. *, *p* < 0.05; **, *p* < 0.01.

**Figure S4.** The IL-8 production was rescued by introduction of a shRNA-resistant KDM4B expression vector in AGS (A) and MKN45 (B) cells. KDM4B^a^ and KDM4B^b^: the #1 and #2 shRNA-resistant KDM4B vectors, respectively.

**Figure S5.** The expression of KDM4A and KDM4B is elevated in gastric cancer. Analysis of KDM4A, KDM4B, and KDM4C mRNA level in gastric cancer tissues from 8 datasets of Oncomine database ([www.oncomine.org](http://www.oncomine.org)).

**Figure S6.** A schematic diagram shows that the histone demethylase KDM4B participates in gastric carcinogenesis via its interaction with the AP-1 transcriptional factor c-Jun. The c-Jun-KDM4B complex contributes to tumorigenesis through upregulation of *IL-8*, *MMP1* and *ITGAV*, which is even more pronounced by challenge with *H. pylori*. Silencing of KDM4B represents a new strategy to suppress the c-Jun/IL-8 pathway.
